# Day/night fluctuations of breast milk bioactive factors and microbiome

**DOI:** 10.3389/fnut.2025.1618784

**Published:** 2025-09-05

**Authors:** Melissa A. Woortman, Haipeng Sun, Jincheng Wang, Filipa Godoy-Vitorino, Angeliz J. Rivera Meléndez, Maribel Campos Rivera, Edna E. Aquino Piñero, Krystin Engelhardt, Lawrence C. Kleinman, Maria G. Dominguez-Bello

**Affiliations:** ^1^Department of Nutritional Sciences, Rutgers University, New Brunswick, NJ, United States; ^2^Department of Biochemistry & Microbiology, Rutgers University, New Brunswick, NJ, United States; ^3^Department of Microbiology, School of Medicine, University of Puerto Rico, San Juan, PR, United States; ^4^School of Nursing, School of Medicine, University of Puerto Rico, San Juan, PR, United States; ^5^Center for Community Outreach for Health Across the Lifespan, Dental and Craniofacial Genomics Core, Medical Sciences Campus, University of Puerto Rico, San Juan, PR, United States; ^6^Department of Pediatrics, Rutgers Robert Wood Johnson Medical School, New Brunswick, NJ, United States; ^7^Rutgers School of Public Health, Piscataway, NJ, United States; ^8^Child Health Institute of New Jersey, New Brunswick, NJ, United States; ^9^Department of Anthropology, Rutgers University, New Brunswick, NJ, United States; ^10^Humans and the Microbiome Program, Canadian Institute for Advanced Research, Toronto, ON, Canada

**Keywords:** breast milk, circadian, milk microbiome, hormones, immune proteins

## Abstract

**Introduction:**

Human breast milk is a sophisticated and complex biological fluid that provides crucial nutritional, immunological, and microbial benefits to infants. Hormones exhibit circadian variations in maternal serum, and understanding these fluctuations in breast milk is crucial for assessing infant maturation. This is particularly relevant when expressed breast milk is fed at a different time from when it was originally produced.

**Methods:**

This study examined 24-h variations in breast milk composition by analyzing samples from 38 lactating mothers at four distinct times of the day. Levels of cortisol, melatonin, immunoglobulin A (IgA), lactoferrin, and oxytocin were quantified using ELISA, and microbiome composition was assessed through 16S rRNA sequencing.

**Results:**

Significant 24-h fluctuations in melatonin and cortisol concentrations were noted, whereas lactoferrin and IgA levels only varied when separating by infant age, maternal BMI, or infant sex. Breast milk microbial composition shifted, with a nocturnal increase in skin-associated bacteria and a diurnal increase in environmental bacteria depending on maternal BMI and infant age. Additionally, milk microbiota alpha diversity increased due to age, but not consistently over all the time points.

**Discussion:**

These differences in 24-h breast milk composition underscore the physiological relevance of maintaining the natural temporal dynamics of breast milk, which may be disrupted when expressed breast milk is fed asynchronously from its time of expression.

## 1 Introduction

Breast milk is frequently acknowledged as the optimal source of infant nutrition, conferring numerous immunological and developmental benefits. Besides providing the proper nutrients to the infant for growth and development, breast milk delivers bioactive compounds such as human milk oligosaccharides (HMOs) (1), hormones (2), and immune factors. Breast milk also harbors its own microbiome which, in combination with many bioactive milk components, aids in inoculating the infant gut (3). Changes in breast milk composition depend on multiple factors, including maternal diet (4), health status (5), infant age (6), geographic location (7), and time of day.

Circadian rhythms regulate many physiological processes in the body and may have a crucial role in the fluctuations of milk components. They are controlled internally through the hypothalamic suprachiasmatic nucleus (SCN), which provides central regulation, and through clock genes in peripheral tissues. Serum hormones, such as melatonin and cortisol, are subject to circadian fluctuations which are driven by internal and external cues. These hormones subsequently influence other circadian processes, such as metabolic pathways and immune functioning (8–11). In neonates, this regulatory system is immature and develops throughout the first year of life (12). The maturation of circadian rhythms is influenced by many factors, such as gestational age and duration of light exposure (13). Circadian rhythms in infants are evident through emerging patterns in temperature, hormone levels, and sleep duration. For example, serum melatonin rhythmicity appears around 2–5 months of age (14), whereas sleep patterns start to emerge around 6 weeks of age and usually normalize by 2 years of age (15).

It has been suggested that bioactive components in breast milk can help provide circadian cues for infants until they can regulate their own rhythms (16). These bioactive factors, such as melatonin and cortisol, often reflect maternal concentrations in serum (17, 18). Other bioactive components, including oxytocin and immunoglobulins, are also influenced by circadian rhythms (19, 20), but their specific variations in breast milk are less well understood (21, 22). Circadian fluctuations of the host influence the gut microbiome through changes in relative abundance (23) and bacteria location in either the intestinal lumen or the epithelial mucosal layer (24), but time-of-day changes in breast milk microbial communities have yet to be established.

These breast milk components are integral to the maturation of biological systems in infants, so investigating their changes in breast milk is crucial. This is particularly relevant in the context of expressing breast milk, as these circadian variations may be negated or misaligned due to asynchrony between the time of milk expression compared to the time of infant feeding or the common practice of pooling expressed milk. This study aimed to investigate the day/night changes in breast milk hormone and immune factor concentrations, as well as microbiome structure, to provide insights into the day/night fluctuations in human milk components. Understanding these changes is important since disruptions in early-life exposure to biologically-timed milk components may contribute to metabolic and immune system dysregulation in infants (25, 26).

## 2 Methods

### 2.1 Study design and participants

To investigate the changes in breast milk throughout the day and night, samples were collected from three separate studies from two sites with similar protocols. The Rutgers Breast Milk Study (Rutgers Arts and Sciences IRB approved, #Pro2018002781) recruited breastfeeding mothers with infants less than one year of age. Exclusion criteria were exclusive formula feeding/lack of breastfeeding, or subjects with infants older than one year. The Puerto Rico Breast Milk Study (University of Puerto Rico, Medical Sciences Campus IRB approved, #B2310120) also recruited breastfeeding mothers with infants less than one year of age but had additional inclusion criteria of the mother and infant being in good health and maternal age of >21 years of age (Supplementary Table S1). Additional exclusion criteria included maternal antibiotic use, illegal drug use/smoking, prepartum obesity, medication use, and not giving birth at term. The Rutgers SARS-CoV-2 and Breast Milk Study (Rutgers University-New Brunswick Health Sciences IRB Protocol #Pro2020002169) recruited mothers who had just given birth at Robert Wood Johnson Medical Center in New Brunswick, NJ, and were either SARS-CoV-2 positive or negative. This study’s inclusion and exclusion criteria included the Puerto Rico study criteria, along with excluding for infant hypoglycemia, infant congenital abnormalities/comorbidities, or admission to the NICU (Supplementary Table S1).

Study subjects in all studies completed a background questionnaire about the health history and demographics of the mother and infant, as well as breastfeeding practices, and provided breast milk samples throughout a 24-h period. The 24-h sampling session was repeated once for the Rutgers and Puerto Breast Milk Studies, approximately one month after the initial sampling, for a total of two sampling sessions per subject whereas the Rutgers SARS-CoV-2 and Breast Milk Study subjects were asked to complete one sampling session. A questionnaire was also administered at the second study visit, when applicable.

### 2.2 Sample collection

For each sampling session, study subjects were provided a sampling kit and instructed to either hand-expressed milk or use their own sterile pump to express milk over the 24-h period at four consecutive time points, which were 6:00 am, 12:00 pm, 6:00 pm, and 12:00 am (Supplementary Figure S1). The subjects could choose which time point to start at and subsequent samples were taken at the next three consecutive time points. At each time point, 10 mL of breast milk was collected in a sterile glass container, aliquoted with a sterile glass pipette into Protein LoBind tubes (Eppendorf AG, Hamburg, Germany), and frozen by the subject at their home. Samples were obtained from the subjects within 48 h of their last sampling and transported on ice for the Puerto Rico Breast Milk Study, or dry ice for the Rutgers studies, back to the laboratory, where they were stored at −80°C until processing.

### 2.3 Hormone and immune protein analysis

Hormones and immune compounds in breast milk that were measured for this study were melatonin, cortisol, oxytocin, IgA, and lactoferrin. Melatonin and cortisol were selected due to their implications with circadian rhythms (27), while oxytocin is connected with suckling and maternal–infant bonding (28). Melatonin and oxytocin also influence intestinal development and gut microbiome dynamics (29, 30), along with IgA and lactoferrin (31). Breast milk hormone and immune factors were determined using compound-specific enzyme-linked immunosorbent assays (ELISAs). Assay validity and cross-reactivity was noted for each ELISA per manufacturer (Supplementary Table S2). To measure melatonin, oxytocin, IgA, and cortisol, thawed breast milk samples were vortexed prior to centrifuging at 1000 x g for 15 min at 4°C in the Eppendorf Centrifuge 5,810 R (Eppendorf AG, Hamburg, Germany) and the aqueous fraction was extracted using a 1 mL syringe. This process was repeated with centrifugation at 3,000 x g for 15 min at 4°C and defatting. The resulting supernatant was utilized to measure hormone and IgA concentrations using the Direct Saliva Melatonin ELISA and Cortisol (Saliva) ELISA kits (Alpco, Salem, NH, United States), as well as the IgA Human SimpleStep ELISA kit (Abcam, Cambridge, MA, United States). For the oxytocin ELISAs, the supernatant obtained was then eluted through HyperSep^™^ C18 Cartridges (Thermo Fisher Scientific, Vilnius, Lithuania) and dried in a vacuum concentrator (Labconco, Kansas City, MO, United States) prior to use in the Oxytocin ELISA (Alpco, Salem, NH, United States). Lactoferrin was measured with whole milk samples that were thawed and vortex briefly prior to using the Lactoferrin ELISA (Alpco, Salem, NH, United States). All ELISAs were performed in triplicate, in accordance with the manufacturer’s protocol, and with standards/controls in each plate for generating standard curves. A 4P log-logistic equation was used to determine concentrations of the compounds based on an optical density (OD) of 450 nm for all the ELISAs except for oxytocin, which was based on an OD of 405 nm.

### 2.4 DNA extraction and sequencing

Details of the DNA extraction and sequencing have been published previously (26). The pellet and 200 μL of supernatant from 1 mL breast milk samples underwent DNA extraction using the Qiagen DNeasy Powersoil Pro Kit (Qiagen, Hilden, Germany). We then PCR-amplified the V4 variable region of the bacterial *16S rRNA* gene using the forward (5’-GTGYCAGCMGCCGCGGTAA-3′) and reverse (5’-GGACTACNVGGGTWTCTAAT-3′) primers in accordance with the Earth Microbiome Project protocol (32) with the Invitrogen Platinum Hot Start Master Mix (Thermo Fischer Scientific, Vilnius, Lithuania). DNA amplicons were quantified with the Qubit dsDNA HS assay kit (Thermo Fisher Scientific, Eugene, OR, United States). Equimolar amounts of the DNA from each sample were pooled and purified using the Qiagen QIAquick PCR purification kit (Qiagen, Hilden, Germany). Sequencing of the samples was subsequently conducted using the Illumina MiSeq platform targeting pair-end 150 reads via GENEWIZ, LLC (South Plainfield, NJ, USA).

### 2.5 Microbiome analyses

Reads were initially analyzed using the QIIME2 pipeline (v2023.5) (33) by demultiplexing and denoising with DADA2 (34) to generate amplicon sequence variants (ASVs), which were compared to the SILVA database r138.2 (35) in order to determine taxonomy. The remainder of the microbiome analyses were conducted following packages available through R v4.3.1 (36). Potential contaminants were screened with the decontam package (37). Rarefaction was set at a sequencing depth of 5,082 to filter out low reads. Shannon Index, Pielou’s Evenness, Faith’s Phylogenetic Diversity, and Observed Features were generated for each sample. Between sample differences were compared using the beta diversity metrics of Bray Curtis Distance, Jaccard Distance, and Weighted and Unweighted Unifrac Distances. Differences in ASV abundance were calculated using Analysis of Compositions of Microbiomes with Bias Correction (ANCOM-BC) (38). Interactions of microbes with each other within their community were determined by a network analysis conducted using the SpiecEasi package (39), setting the correlation cutoff to 0.3.

### 2.6 Statistical analyses

Data from previous studies indicated that 20–40 subjects provided sufficient power to see statistically significant differences in breast milk component concentrations over 24–48 h (21, 40, 41). Hormone and immune protein concentrations, as well as microbiome alpha diversity, were measured for normality using the Shapiro Wilk test. For normally distributed data, Student t-test and analysis of variance (ANOVA) were utilized. When data was not normally distributed, Wilcoxon Rank Sum and Kruskal Wallis tests were used to identify differences between time points. Beta diversity was compared using permutational multivariate ANOVA (PERMANOVA) (42). A significance level of 0.05 was used unless otherwise noted.

## 3 Results

### 3.1 Subject participation and characteristics

This study recruited subjects from three different projects that took place in New Brunswick, NJ or in Puerto Rico, for a total of 43 participants who met the inclusion criteria (Supplementary Table S1; Supplementary Figure S2). Out of those 43 subjects, two became ineligible due to illness post-enrollment and three subjects were unable to provide sufficient samples and therefore were not included in the analyses. From the 38 active participants, 21 provided samples on two different days (about 1 month apart), and 17 provided samples once (Supplementary Figures S1, S2). In total, there were 59 sampling instances and 236 samples included in the analyses (Supplementary Table S3). The demographic characteristics of the participants are shown in Table 1; in short, 58% of the participants were white, 18% were classified as having an obese body mass index (BMI), 79% delivered vaginally, 45% directly breastfed with feeding some expressed breast milk, and 71% had not yet started providing solid foods to their baby. At the time of the first sample, 55% of the infants were aged 0–3 months, 18% were 3–6 months, 18% were 6–9 months, and 8% were 9–12 months (Table 1). Unless indicated, the demographic characteristics were not included in the statistical models.

**Table 1:** Baseline subject characteristics.

Maternal characteristics (*n* = 38)	*n*	%
Race/ethnicity		
White	22	58
Hispanic	7	18
Asian/Pacific Islander	6	16
Black/African American	3	8
Marital status		
Married	35	92
Single	3	8
Highest degree earned		
High school diploma/GED	3	8
Associate degree	4	11
Bachelor’s degree	12	32
Master’s degree	12	32
PhD	3	8
Medical doctorate	4	11
Employment status		
Full-time	21	55
Part-time	6	16
Not employed	10	26
Full-time student	1	3
Gross annual household income		
Less than $50,000	8	21
$50,000 or more	30	79
Age range (years)		
21–30	7	18
31–40	31	82
BMI		
Normal (18.5–24.9 kg/m^2^)	18	47
Overweight (25–29.9 kg/m^2^)	12	32
Obese (>30 kg/m^2^)	7	18
Unknown	1	3
Intrapartum antibiotic use		
Yes	3	8
No	35	92
Postpartum antibiotic use		
Yes	4	11
No	34	89
Antibiotic use (mother or infant)		
Yes	8	21
No	30	79

Infant characteristics (*n* = 38)	*n*	%
Gestational age		
36–38 weeks	2	5
38–40 weeks	17	45
40 + weeks	18	47
Unknown	1	3
Sex		
Female	21	55
Male	17	45
Age range (months)		
0–3	21	55
3–6	7	18
6–9	7	18
9–12	3	8
Breastfeeding (BF) mode		
Direct BF only	15	39
Direct BF/formula	2	5
Direct BF/expressed milk	17	45
Direct BF/expressed milk/formula	4	11
Consuming solid foods		
Yes	11	29
No	27	71
Birth mode		
Vaginal delivery	30	79
Cesarean section	8	21
Number of siblings		
0	12	32
1	18	47
2	7	18
3	1	3
Antibiotic use		
Yes	1	3
No	37	97
Weight-for-age range (WHO z-score)		
Above 3	1	3
2 through 3	1	3
1 through 2	1	3
0 through 1	13	34
−1 through 0	13	34
−2 through −1	2	5
−3 through −2	1	3
Missing	6	16

### 3.2 Hormonal and immune protein fluctuations

**Figure 1:**
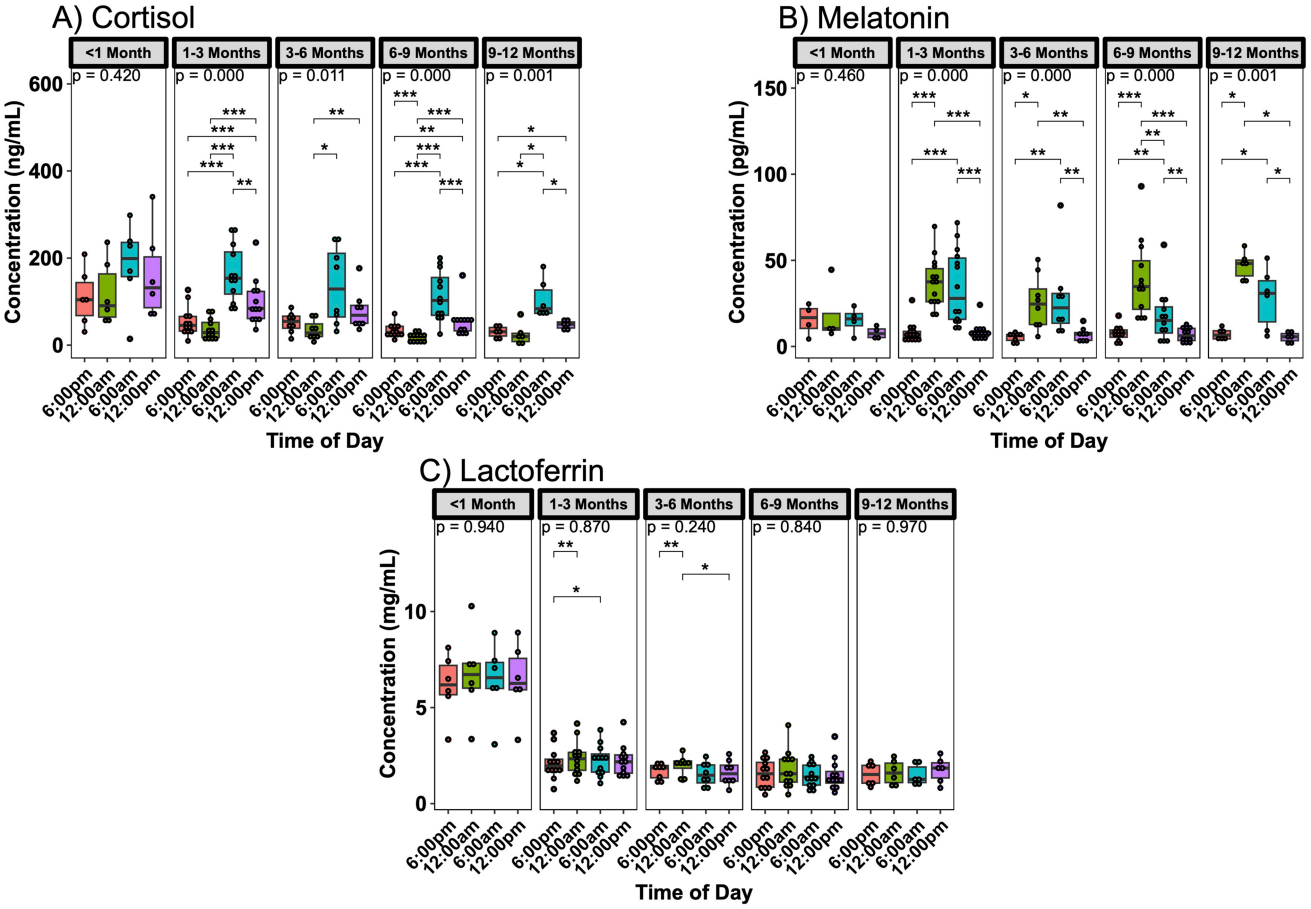
Infant age influence on day/night variations in breast milk components. **(A)** Cortisol, **(B)** melatonin, and **(C)** lactoferrin concentration changes over 24 h are more pronounced in the subjects with 1–3 month old infants. Analysis for cortisol and lactoferrin included 6 sampling instances <1 month, 12 sampling instances in 1–3 months, 8 instances in 3–6 months, 12 instances in 6–9 months, and 6 instances for 9–12 months of age. Analysis for melatonin included 4 sampling instances <1 month, 14 sampling instances in 1–3 months, 8 instances in 3–6 months, 12 instances in 6–9 months, and 6 instances for 9–12 months of age. Group comparisons performed with Kruskal-Wallis, comparisons between two time points performed with Wilcoxon Rank Sum Test. * *p* < 0.05, ** *p* < 0.01, *** *p* < 0.001, **** *p* < 0.0001.

Analysis of breast milk samples revealed significant day/night fluctuations in key bioactive components. Strong fluctuations were observed with melatonin and cortisol, with melatonin peaking at midnight and cortisol peaking at 6:00 am (Supplementary Figures S3A,B). These fluctuations were also present when stratifying by age, except the subjects with infants <1 month of age did not exhibit significant fluctuations (Figures 1A,B). Oxytocin, IgA, and lactoferrin did not show significant concentration variations based on the time of day (Supplementary Figures S3C–E). While IgA and oxytocin did not show significant variations when separated by age (Supplementary Figure S4), lactoferrin exhibited significant fluctuations between time points for mothers with infants 1–6 months of age (Figure 1C). Differences between samples obtained from the same study participants in two different instances (about 1 month apart), were not different in their circadian fluctuations.

Conversely, differences between infant ages were noted for all compounds when looking at each time point separately. Cortisol had the least number of significant differences by age at its peak at 6:00 am (Supplementary Figure S5A), where melatonin had differences at its peak hours but not when breast milk concentration was low (Supplementary Figure S5B). Oxytocin only showed differences at 6:00 am only between the 0–3- and 3–6-month-old groups (Supplementary Figure S5C). IgA and lactoferrin concentrations were consistently higher in infants aged <1 month across all time points (Supplementary Figures S5D,E).

**Figure 2:**
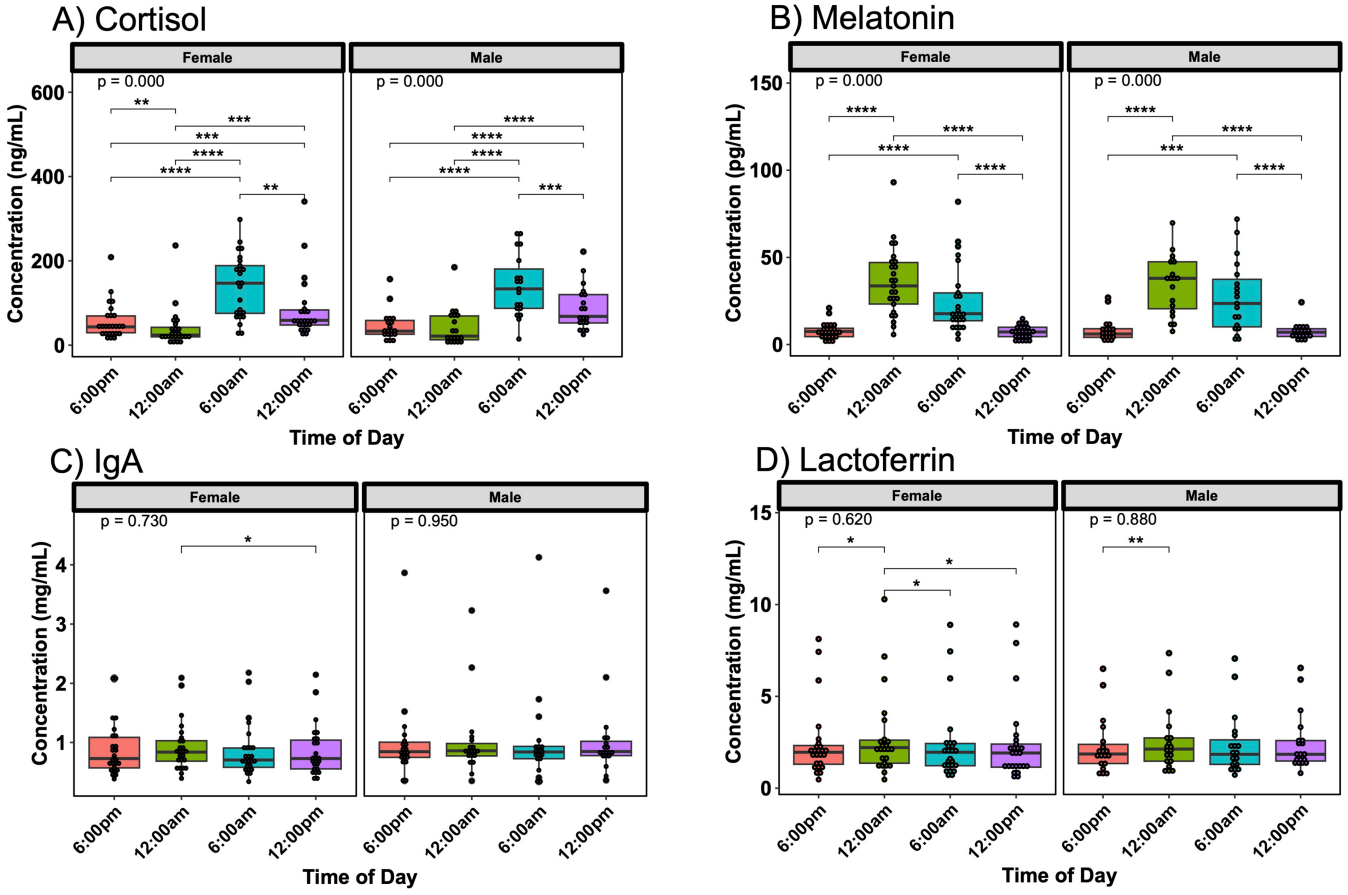
Day/night variations in breast milk hormones and immune proteins by infant sex. Concentration fluctuations of **(A)** cortisol and **(B)** melatonin were similar when separated by infant sex, whereas **(C)** IgA and **(D)** lactoferrin fluctuations differed based on infant sex. Analysis for cortisol, melatonin, IgA, and lactoferrin included 25 sampling instances for subjects with female infants and 19 instances for subjects with male infants. Group comparisons performed with Kruskal-Wallis, comparisons between two time points performed with Wilcoxon Rank Sum Test. * *p* < 0.05, ** *p* < 0.01, *** *p* < 0.001, **** *p* < 0.0001.

Cortisol, melatonin IgA, and lactoferrin exhibited some differences in significance across various time points when divided by infant sex, though the hormone fluctuations were similar between mothers with female or male infants (Figure 2). Mothers of female infants showed the most significant variations of concentration of lactoferrin in milk based on the time of day when compared to those of male infants. No differences were noted in oxytocin based on infant sex (Supplementary Figure S6).

**Figure 3:**
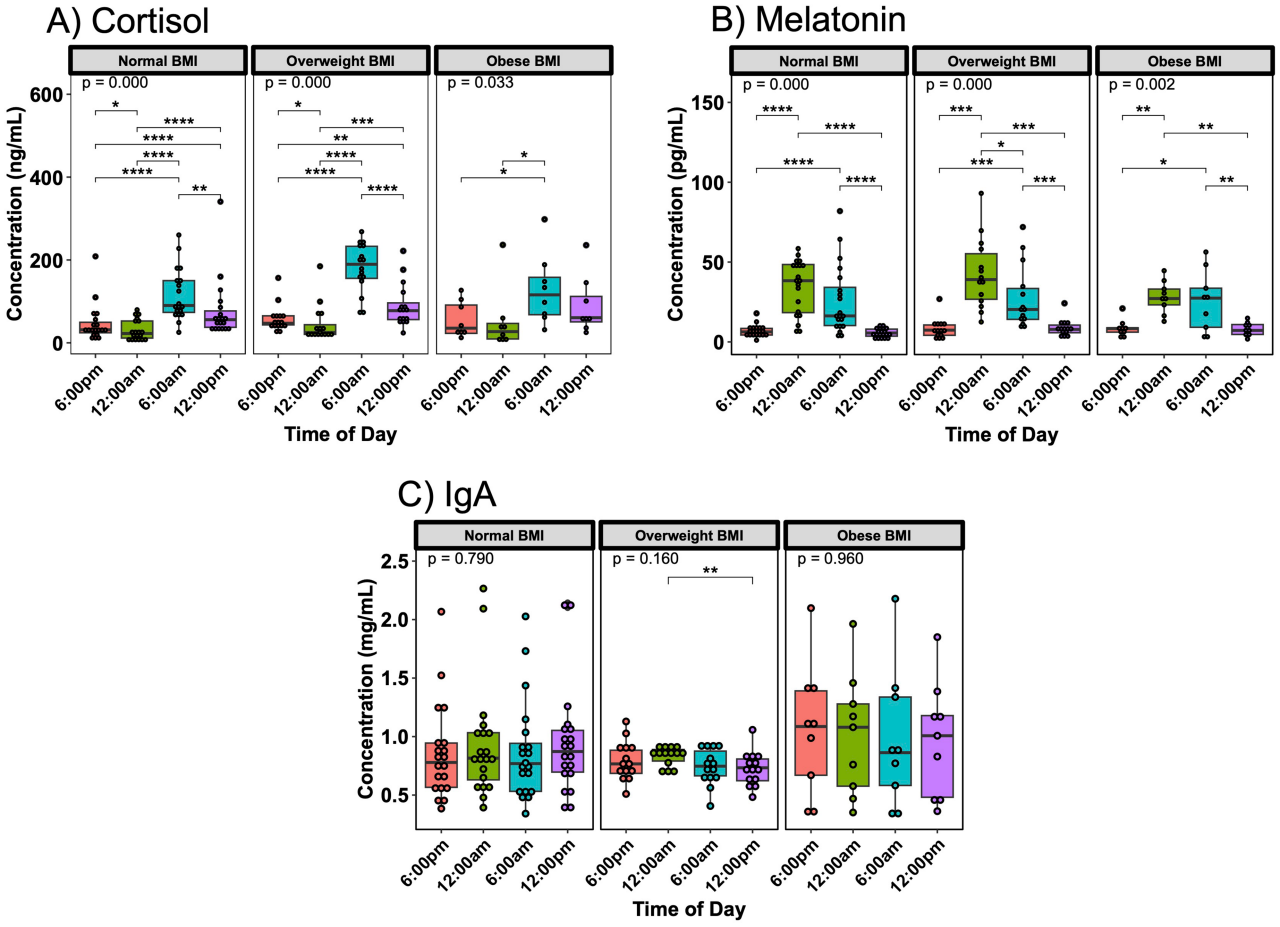
Day/night variations in breast milk hormones and IgA vary based on maternal BMI. In **(A)** cortisol and **(B)** melatonin, subjects with an obese BMI at time of sampling showed fewer differences in 24 h concentration in breast milk. For IgA **(C)**, subjects with an overweight BMI had significantly difference breast milk concentrations between midnight and noon. Analysis for cortisol included 19 sampling instances for normal BMI subjects, 16 sampling instances for overweight BMI subjects, and 8 instances for obese BMI subjects. Analysis for melatonin and IgA included 20 sampling instances for normal BMI subjects, 14 sampling instances for overweight BMI subjects, and 9 instances for obese BMI subjects. Group comparisons performed with Kruskal-Wallis, comparisons between two time points performed with Wilcoxon Rank Sum Test. * *p* < 0.05, ** *p* < 0.01, *** *p* < 0.001, **** *p* < 0.0001.

Interestingly, maternal BMI appeared to correlate with the intensity of breast milk hormone fluctuations. Mothers with an obese BMI exhibited blunted variations in breast milk cortisol and melatonin (Figures 3A,B). IgA concentrations also showed variations based on time of day in overweight mothers, but not with normal or obese maternal BMI (Figure 3C). Oxytocin and lactoferrin did not exhibit any significant 24 h variation related to maternal BMI (Supplementary Figure S7). Looking at the different time points separately, only cortisol showed differences between subjects with a normal or overweight BMI at two time points (Supplementary Figure S8A), while the other hormones and immune compounds did not differ (Supplementary Figures S8B–E).

### 3.3 Microbiome fluctuations

Overall measures of alpha diversity (Shannon Entropy, Pielou’s Evenness, Faith’s Phylogenetic Diversity, Observed Features; Supplementary Figure S9) and beta diversity (Bray Curtis, Unweighted Unifrac, Jaccard, Weighted Unifrac; Supplementary Figure S10) did not show significant differences across the 24-h period or between comparisons of time points separately. Stratifying based on infant age revealed a change in alpha diversity in the youngest age group, but only between two time points (Supplementary Figure S11). Alpha diversity did increase by age of the infant, primarily between the infants less than one month of age and the other age groups. However, this effect was not seen at the 6:00 am time point with all the alpha diversity metrics (Supplementary Figure S12).

**Figure 4:**
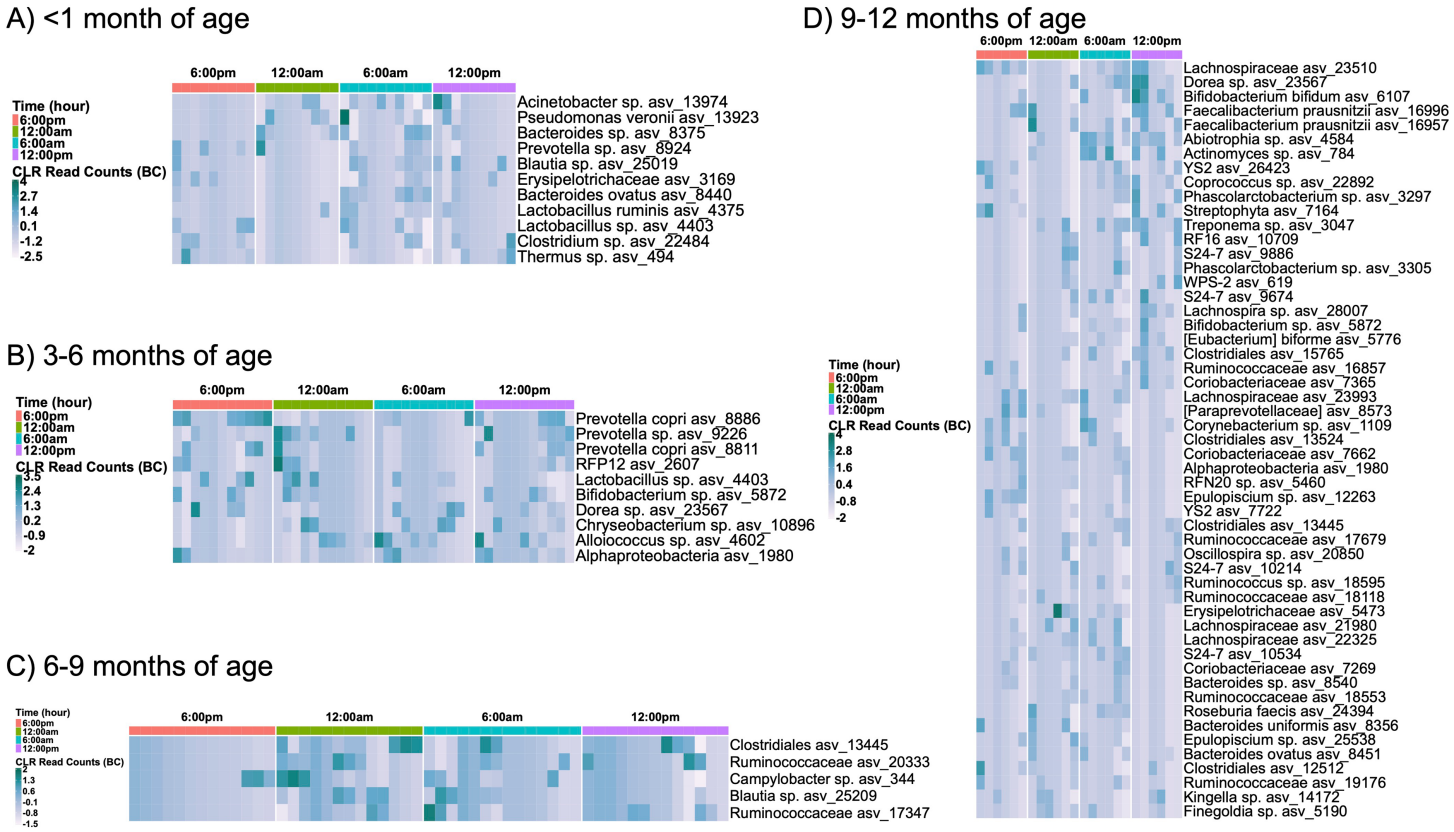
Day/night variations in ASV enrichment separated by infant age. Only significantly different (*p* < 0.05) ASV abundances shown for subjects with infants **(A)** < 1 month, **(B)** 3–6 months, **(C)** 6–9 months, or **(D)** 9–12 months of age; no significant differences were noted in ASV enrichment for subjects with infants aged 1–3 months. Microbiome analysis included 9 sampling instances <1 month, 16 sampling instances in 1–3 months, 14 instances in 3–6 months, 14 instances in 6–9 months, and 6 instances for 9–12 months of age. Comparisons of ASV enrichment performed using ANCOM with Bias Correction (ANCOM-BC). ASV = amplicon sequence variant.

Enrichment of ASVs also changed over a 24 h period when separated by infant age, except for the 1-3-month-old infant group (Figure 4). Interestingly, breast milk exhibited a nocturnal enrichment of skin-associated bacteria and a diurnal enrichment of environmental bacteria. An increase in skin-associated ASVs, including *Prevotella* sp. and *Finegoldia* sp., were observed at the 6:00 pm and midnight time points, while soil-associated bacteria were more abundant in the morning samples. These included Alphaproteobacteria, *Chryseobacterium* sp., Clostridiales, the YS2 order of Cyanobacteria, Streptophyta, and Lachnospiraceae. Infant sex did not correlate with differences in breast milk alpha diversity (Supplementary Figure S13).

Maternal BMI did not appear to impact changes in alpha diversity over 24 h (Supplementary Figure S14), but differences in ASV abundances were noted in mothers with obese BMIs at various sampling times, a pattern not observed among normal or overweight BMI mothers. Enrichment of *Prevotella* sp. was seen in the evening and night hours, while Clostridiales was noted to be enriched in the morning (Supplementary Figure S15).

Bacterial network analysis showed that there were no significant temporal variations in microbial community structure (Supplementary Figure S16), but it showed time-of-day variations in the most interconnected ASVs. At 6:00 am, *Bacteroides* sp., *Prevotella* sp., and *Prevotella copri* were the most interconnected ASVs, while *Stenotrophomonas geniculata* had the most connections at 12:00 pm. Comomonadaceae was noted with the most connections at 6:00 pm, and the most interconnected at midnight were *Bifidobacterium* sp., *Bacteroides* sp., and *Megasphaera* sp.

## 4 Discussion

### 4.1 Hormones and immune proteins

The results of this study show clear changes in breast milk bioactive components over a 24 h period, particularly with cortisol and melatonin. Additionally, changes in bioactive components are associated with infant age and maternal BMI. These findings emphasize the importance of preserving natural temporal dynamics in breastfeeding, as disruptions may impact infant development, metabolism, and immune regulation.

The patterns of melatonin and cortisol observed in this study are consistent with prior findings (17, 22), reinforcing the role of breast milk as a time-sensitive biological signal that may aid in the establishment of infant circadian rhythms. Notably, cortisol levels decreased with infant age, whereas melatonin fluctuations became more pronounced over time, suggesting age-dependent regulatory changes in breast milk composition (16, 18), and the physiological relevance for the infant is unclear. One possible explanation for these changes is that melatonin and cortisol enter the mammary epithelia through diffusion (17, 43), so stabilization of maternal circadian rhythms during the postpartum period (44) could change the concentrations of these hormones in maternal circulation and therefore in breast milk as well. Unlike melatonin and cortisol, oxytocin levels remained stable throughout the day and night. However, as direct breastfeeding induces oxytocin peaks in maternal circulation (45), expressed milk may lack the synchrony of oxytocin secretion associated with hearing their infant cry and suckling (46), although oxytocin responses to these actions may only last for several minutes (45). This raises concerns about whether feeding expressed milk at different times than when it was produced might affect maternal–infant bonding and stress regulation. Further research is needed to explore these potential effects.

Breast milk composition dynamically changes as the infant develops. In this study, cortisol, IgA, and lactoferrin were highest in early infancy (<1 month) and declined over time. This aligns with previous research showing that cortisol concentrations decrease with infant age (18). Lactoferrin and IgA were higher in early lactation, consistent with prior reports (6, 47, 48). Given IgA and lactoferrin’s role in immune defense and microbiome development, its age-related decline may reflect a shift in infant immune needs as gut colonization stabilizes (49, 50). The higher levels of IgA and lactoferrin in younger infants further support the idea that breast milk adapts to support immune system maturation (6, 51).

Sex-based differences observed in the current study, with mothers of female infants showing stronger day/night variations in IgA and lactoferrin, have not been reported previously (52–54), but sex-specific circadian patterns in breast milk remain largely unexplored. Prior studies have shown differences based on infant sex for breast milk lactoferrin (55) but not for IgA concentration (4) in breast milk. Some sex differences have been noted with circadian rhythm development in infants (56), so it is possible that these differences are detected through maternal–infant feedback (57). Future studies should investigate whether hormonal or immune fluctuations in breast milk are linked to sex-specific infant development.

Based on our findings, maternal BMI was associated with circadian variations in breast milk hormones. Mothers with an obese BMI exhibited blunted fluctuations in cortisol and melatonin, with less pronounced differences between peak and low levels compared to normal-weight and overweight mothers (58, 59). Since cortisol plays a key role in metabolic regulation, these findings suggest a potential long-term metabolic impact on infants consuming milk from mothers who are categorized as obese (60). However, oxytocin and lactoferrin were not significantly affected by maternal BMI, indicating selective modulation of certain bioactive components (48).

### 4.2 Breast milk microbiome

Microbiome analysis revealed stable alpha- and beta-diversity across the 24 h period, suggesting that while breast milk composition is regulated temporally, its overall microbial diversity remains stable. However, when considering infant age and maternal BMI, specific bacterial ASVs fluctuated. A nighttime increase in skin-associated bacteria [e.g., *Prevotella* sp. and *Finegoldia* sp. (61)] and a daytime increase in environmental bacteria [e.g., *Chryseobacterium* sp. (62), Clostridiales, and the YS2 order of Cyanobacteria (63)] was observed, possibly reflecting differences in maternal activity patterns, feeding behavior, and infant oral contact (25, 26). Oral microbes from the infant’s mouth often co-inoculate with the maternal breast (64, 65), so changing the frequency and duration of this exposure could alter the milk microbiome composition (66). Notably, in mothers with an obese BMI, these microbial fluctuations were more pronounced, suggesting potential metabolic influences on the breast milk microbiota (67).

Interestingly, network analysis identified different bacterial ASVs as key players at specific times of day. For example, *Bacteroides* sp. and *Prevotella* sp. were the most centrally connected in the early morning, while *Bifidobacterium* sp. and *Megasphaera* sp. where the most connected ASVs at midnight. This suggests that while total microbial diversity remains stable, there are distinct temporal shifts in microbial composition and interactions, which could influence infant gut colonization and immune priming (67).

### 4.3 Limitations

While our study sought to discern differences in breast milk microbiome and other bioactive components over a 24-h period, there are some limitations to note. In terms of maternal BMI, weights and heights were self-reported and the weight requested was at the time of sampling. Since weight often fluctuates in the postpartum period (68), utilizing pre-pregnancy weight, or usual body weight might yield different results. Additionally, this study did not compare demographic characteristics of mothers and infants due to lack of power. Since milk microbiome and bioactive factors vary based on geographic location (69), delivery mode (70, 71), and infant anthropometrics (72), comparing changes based on time of day according to these factors will be important for future studies.

### 4.4 Implications and future directions

These findings have important implications for breastfeeding practices, particularly for mothers who express and feed milk via bottle at a time asynchronous from when it was produced. Since breast milk composition is naturally timed to support infant circadian rhythms, altering feeding schedules could disrupt biologically timed cues for infant sleep, metabolism, and immune development (43, 73, 74).

From a maternal well-being perspective, modern breastfeeding practices—such as expressing milk while working—could impact maternal stress, hormone secretion, and infant-mother interactions (75). This underscores the need for evidence-based guidelines on feeding expressed milk that consider both maternal and infant circadian biology (27, 76).

Future research is needed to better understand the long-term effects of feeding expressed milk asynchronously on infant circadian rhythm development. The present study confirms that breast milk composition relates to circadian rhythms, infant age, and maternal BMI. These findings highlight the critical role of biologically timed milk components in infant development and suggest that the timing of milk feeding may be an overlooked factor in infant health outcomes.

## Data Availability

The names of the repository/repositories and accession number(s) can be found here: https://www.ncbi.nlm.nih.gov/, PRJNA1250551.
