# Supplementary material for: Day/night fluctuations of breast milk bioactive factors and microbiome
Source: Front Nutr. 2025 Sep 5;12:1618784. doi: 10.3389/fnut.2025.1618784 (PMC12446335; doi:10.3389/fnut.2025.1618784)
Supplement: Supplementary file 1 [file Data_Sheet_1.docx]

Supplementary Material

# Supplementary Figures and Tables

## Supplementary Tables

**Table S1: Puerto Rico and Rutgers SARS-CoV-2 Breast Milk Studies- subject inclusion and exclusion criteria.**

|  | Inclusion Criteria | Exclusion Criteria |
| --- | --- | --- |
| Both Studies | Mother/infant in good health | <38 or >42 weeks gestation |
|  | Currently/plan on breastfeeding | Exclusive or supplemental formula feeding |
|  | Agreeable to study procedures | Breastfeeding cessation |
|  |  | Illegal drug use/smoking |
|  |  | Maternal BMI >30 kg/m^2^ pre-pregnancy |
|  |  | Maternal/infant health issues |
| Puerto Rico Breast Milk Study | Have an infant <1 year of age | <20 years of age |
|  | >21 years of age | >4 alcoholic beverages/week |
|  |  | Antibiotic or long-term medication or use during or after pregnancy |
| Rutgers SARS-CoV-2 and Breast Milk Study | Gave birth at Robert Wood Johnson- University Hospital | Infant hypoglycemia |
|  | English-speaking | Infant admission to NICU |
|  | >38 weeks gestation | Infant congenital abnormalities |

**Table S2: ELISA Validity and Cross-Reactivity.** CV = coefficients of variances, CR = Cross-Reactivity

| Compound | Intra-Assay Precision | | | Inter-Assay Precision | | Cross-Reactivity | |
| --- | --- | --- | --- | --- | --- | --- | --- |
|  | Concentration | CV (%) | | Concentration | CV (%) | Compound | CR (%) |
| Cortisol | 6.6 ng/mL | 10.3 | | 6.3 ng/mL | 9.8% | Cortisol | 100 |
|  |  |  |  |  |  | Prednisolone | 13.6 |
|  |  |  |  |  |  | Corticosterone | 7.6 |
|  | 24.8 ng/mL | 8.0 | | 23.7 ng/mL | 8.7% | Deoxycorticosterone | 7.2 |
|  |  |  |  |  |  | Progesterone | 7.2 |
|  |  |  |  |  |  | Cortisone | 6.2 |
|  | 52.4 ng/mL | 6.5 | | 51.8 ng/mL | 6.5% | Deoxycortisol | 5.6 |
|  |  |  |  |  |  | Pednisone | 5.6 |
|  |  |  |  |  |  | Dexamethasone | 1.6 |
| Melatonin | 1.7 pg/mL | 10.8 | | 2.1 pg/mL | 12.7 | NA | NA |
|  | 5.1 pg/mL | 6.1 | | 4.9 pg/mL | 7.6 |  |  |
|  | 33.2 pg/mL | 8.7 | | 14.7 pg/mL | 13.0 |  |  |
| Oxytocin | 39.9 pg/mL | 12.6 | 47.0 pg/mL | | 20.9 | Mesotocin | 7.00 |
|  |  |  |  |  |  | Arg8-Vasotocin | 7.50 |
|  |  |  |  |  |  | Ser4,Ile8-Oxytocin | <0.02 |
|  |  |  |  |  |  | THR | <0.02 |
|  | 121.4 pg/mL | 10.2 | 145.1 pg/mL | | 16.5 | Growth Hormone | <0.02 |
|  |  |  |  |  |  | Tocinoic Acid | <0.02 |
|  |  |  |  |  |  | Meanostatin | <0.02 |
|  |  |  |  |  |  | Somatostatin | <0.02 |
|  | 363.7 pg/mL | 13.3 | 397.2 pg/mL | | 11.8 | Met-Enkephalin | <0.02 |
|  |  |  |  |  |  | VIP | <0.02 |
|  |  |  |  |  |  | Lys8-Vasopressin | <0.02 |
|  |  |  |  |  |  | Arg8-Vasopressin | <0.02 |
|  |  |  |  |  |  | α−ANP | <0.02 |
| IgA | NA | 4.12 | | NA | 5.92 | No significant CR | NA |
| Lactoferrin | NA | <10 | | NA | <10 | NA | NA |

**Table S3: Number of subjects and breast milk samples across all studies**

| Study | Total subjects | 1 sampling instance | 2 sampling instances | Total sampling instances | Total Samples |
| --- | --- | --- | --- | --- | --- |
| Rutgers Breast Milk Study | 24 | 8 | 16 | 40 | 160 |
| Rutgers COVID Study | 9 | 9 | 0 | 9 | 36 |
| Puerto Rico Breast Milk Study | 5 | 0 | 5 | 10 | 40 |
| Totals | 38 | 17 | 21 | 59 | 236 |

## Supplementary Figures


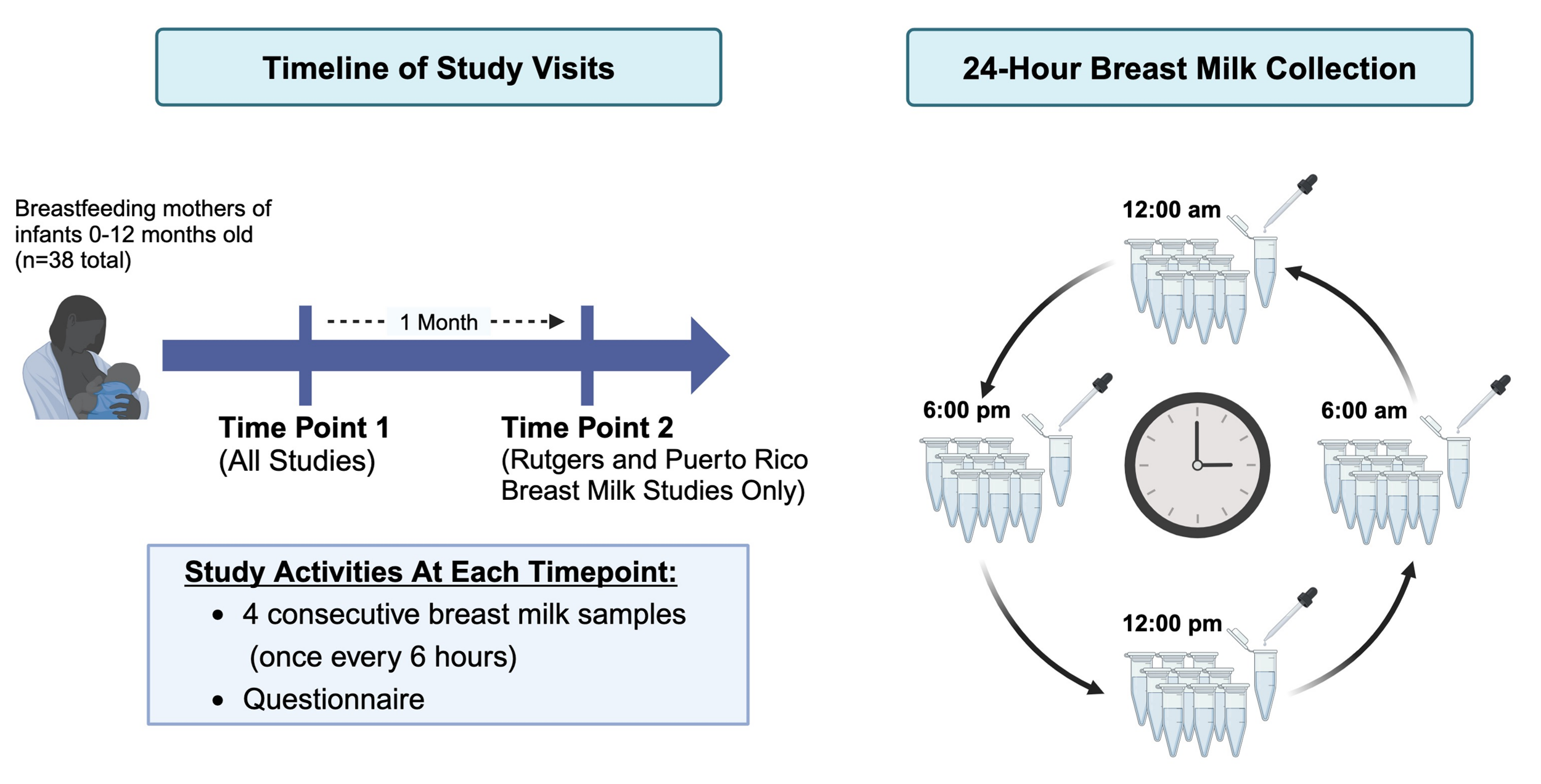
**Figure S1: Experimental Design.** Created in <https://BioRender.com>


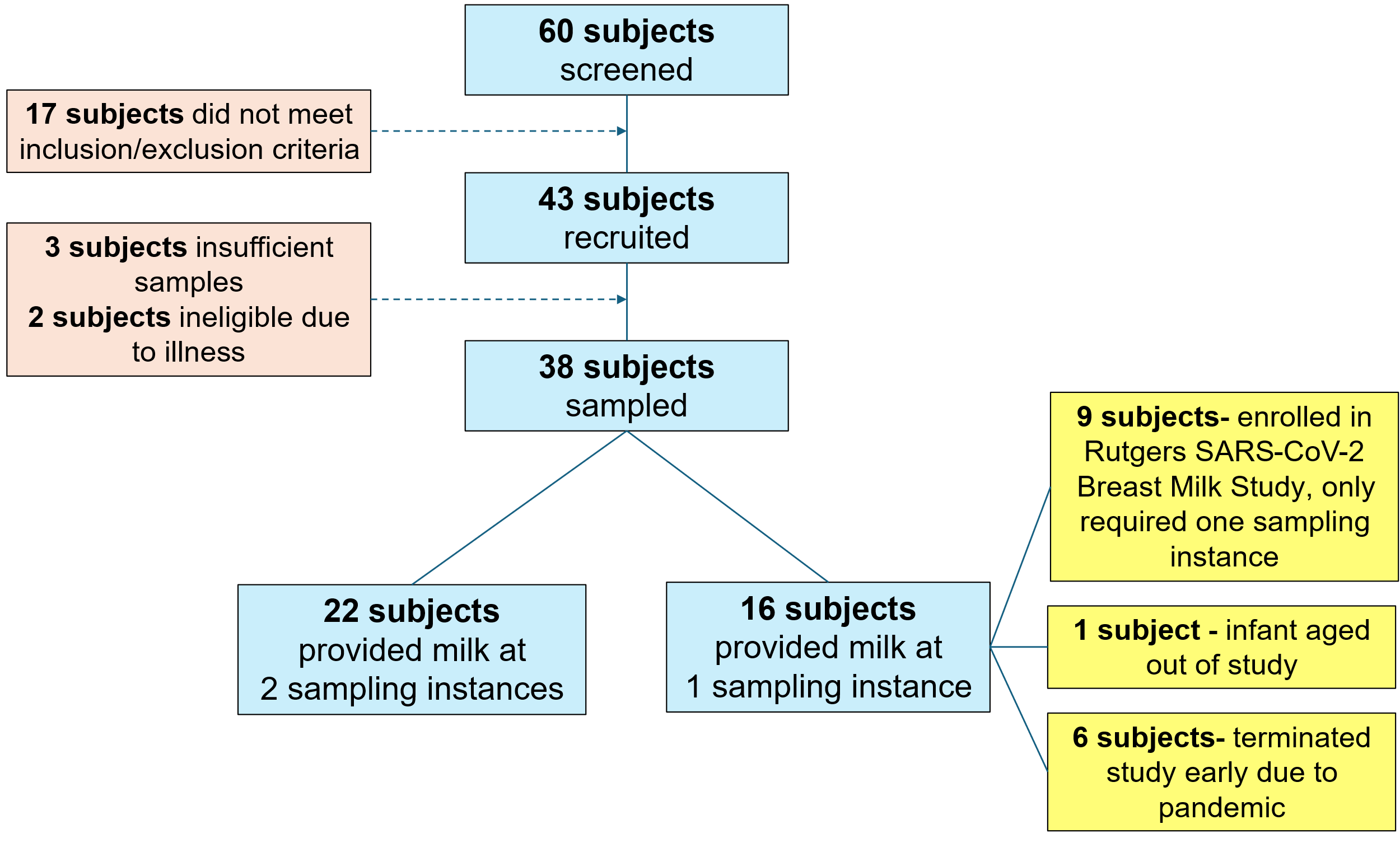
**Figure S2: Subject enrollment flow chart.**


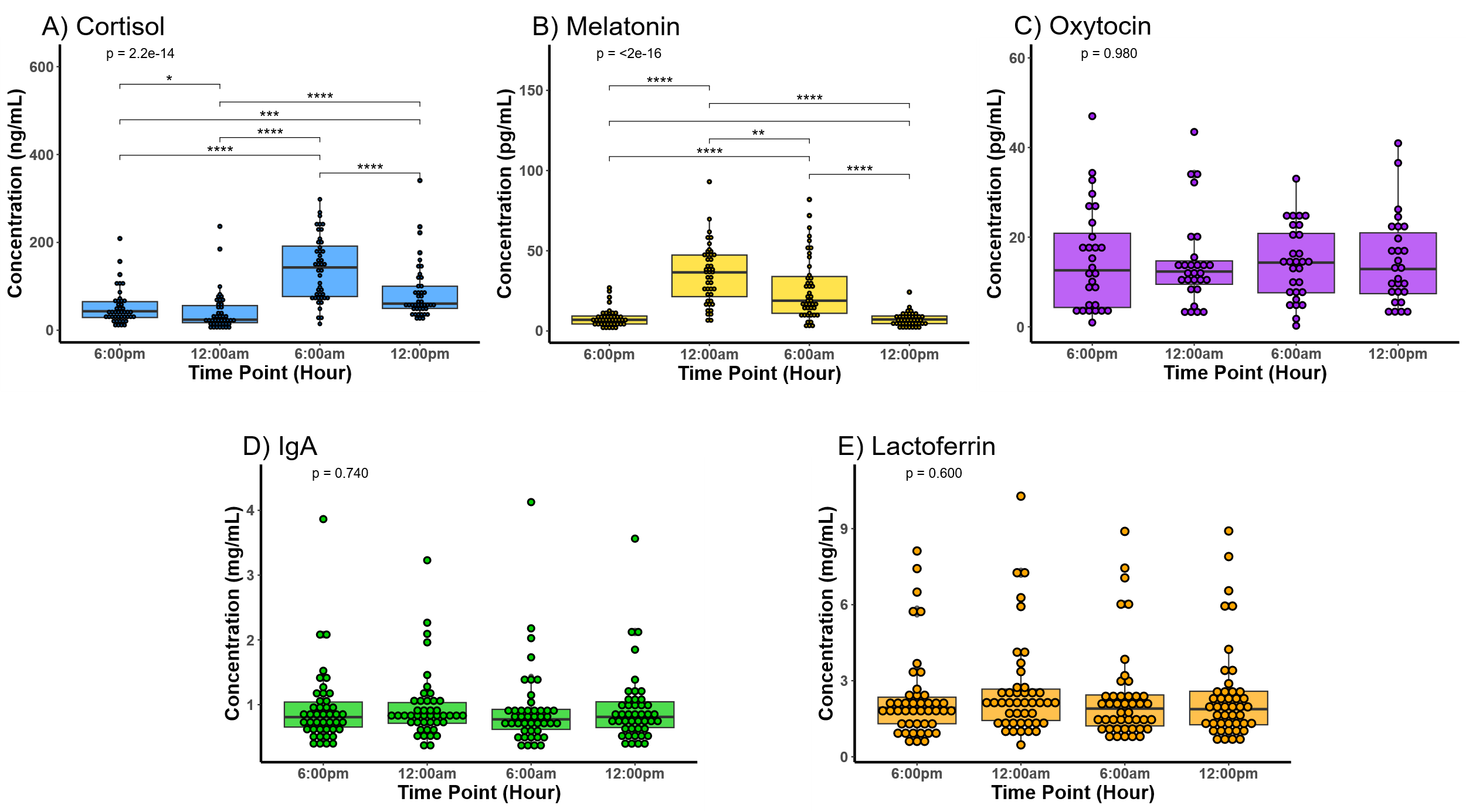
**Figure S3: Concentration of breast milk hormones varies by time of day.** (A) Cortisol peaked at the 6:00 am time point and (B) melatonin peaked at the12:00 am time point, whereas (C) oxytocin, (D) IgA, and (E) lactoferrin did not exhibit temporal fluctuations. Analysis included 44 sampling instances from 30 subjects. Group comparisons performed with Kruskal-Wallis, comparisons between two time points performed with Wilcoxon Rank Sum Test. * p < 0.05, ** p < 0.01, *** p < 0.001, **** p< 0.0001.


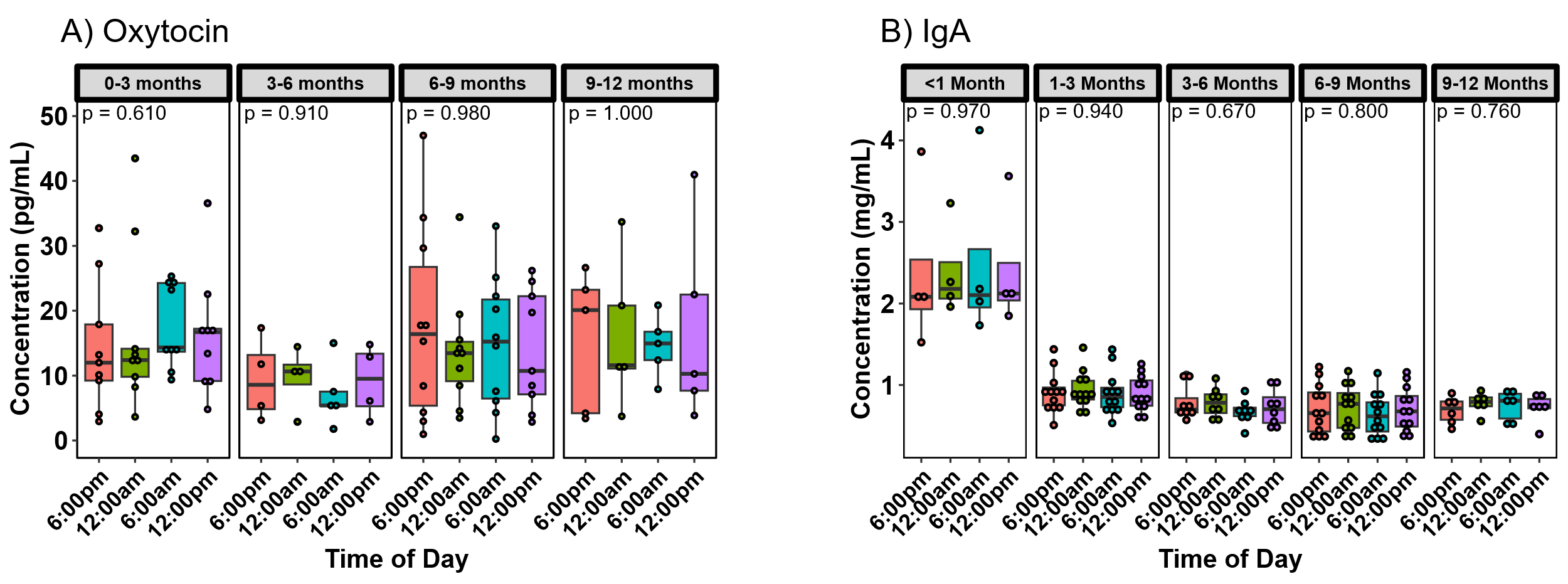


**Figure S4: Concentrations of breast milk oxytocin and IgA do not exhibit fluctuations over a 24-hour period when separated by infant age.** No significant differences noted for (A) oxytocin or (B) IgA over a 24-hour period when separating by infant age (p > 0.05). Analysis for oxytocin included 0 sampling instances <1 month, 9 sampling instances in 1-3 months, 5 instances in 3-6 months, 11 instances in 6-9 months, and 5 instances for 9-12 months of age. Analysis for IgA included 4 sampling instances <1 month, 14 sampling instances in 1-3 months, 8 instances in 3-6 months, 12 instances in 6-9 months, and 6 instances for 9-12 months of age. Group comparisons performed with Kruskal-Wallis, comparisons between two time points performed with Wilcoxon Rank Sum Test.

**
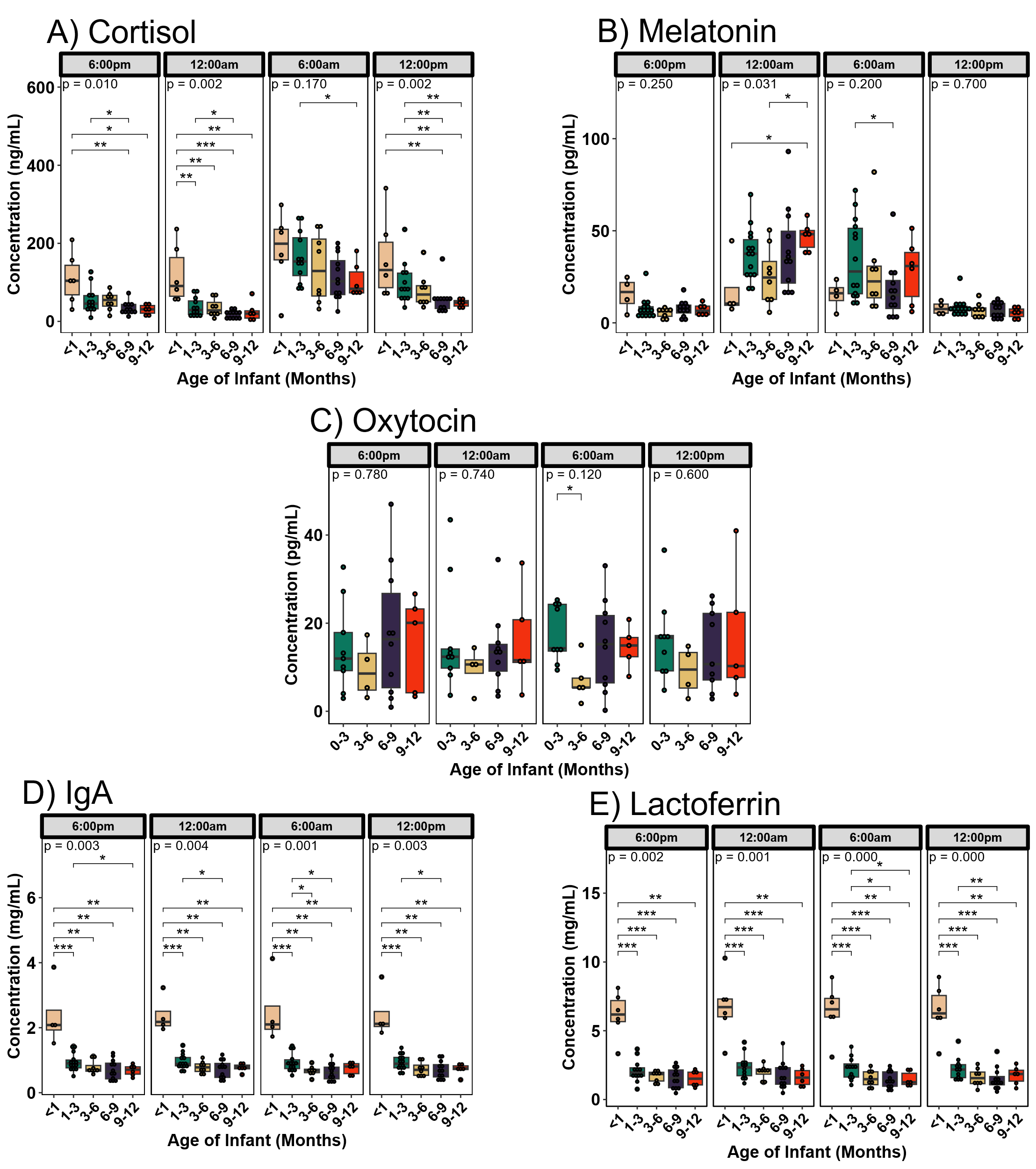
Figure S5: Concentrations of hormones and immune proteins in breast milk vary based on infant age at different times in a 24-hour period.** (A) Cortisol, (B) melatonin, and (C) oxytocin concentrations in breast milk did not show consistent differences between infant ages over the course of a 24-hour period, whereas (D) IgA and (E) lactoferrin concentrations were markedly higher across all time points for mothers with infants <1 month of age. Analysis for cortisol and lactoferrin included 6 sampling instances <1 month, 12 sampling instances in 1-3 months, 8 instances in 3-6 months, 12 instances in 6-9 months, and 6 instances for 9-12 months of age. Analysis for melatonin and IgA included 4 sampling instances <1 month, 14 sampling instances in 1-3 months, 8 instances in 3-6 months, 12 instances in 6-9 months, and 6 instances for 9-12 months of age. Analysis of oxytocin included 0 sampling instances <1 month, 9 sampling instances in 1-3 months, 5 instances in 3-6 months, 11 instances in 6-9 months, and 5 instances for 9-12 months of age. Group comparisons performed with Kruskal-Wallis, comparisons between two time points performed with Wilcoxon Rank Sum Test. * p < 0.05, ** p < 0.01, *** p < 0.001, **** p< 0.0001.


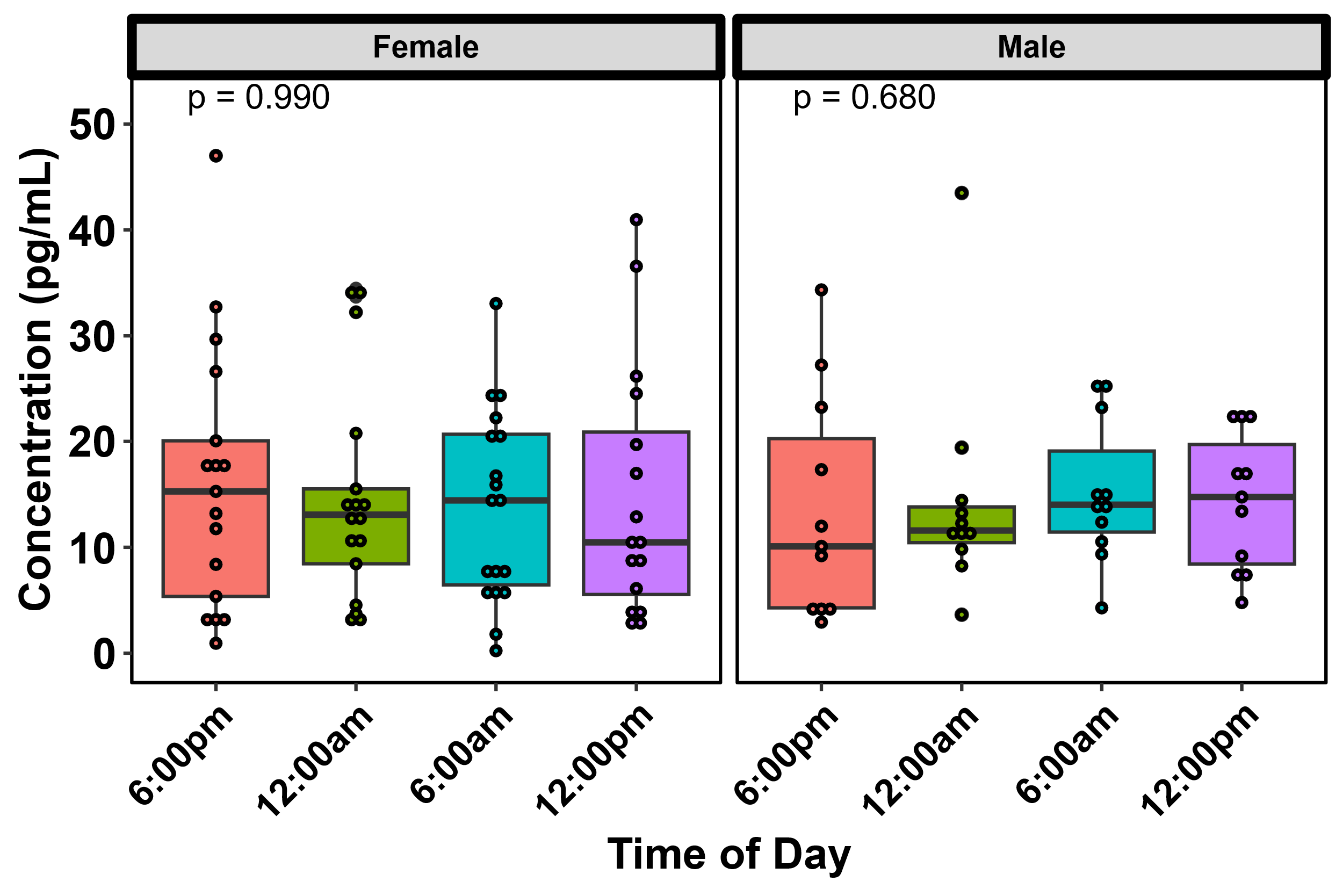
**Figure S6: Variations of breast milk oxytocin concentrations did not differ when separated by infant sex.** No significant differences noted for oxytocin in breast milk over a 24-hour period, separated by sex of the infant (p > 0.05). Analysis for oxytocin included 19 sampling instances for subjects with female infants and 12 instances for subjects with male infants. Group comparisons performed with Kruskal-Wallis, comparisons between two time points performed with Wilcoxon Rank Sum Test.


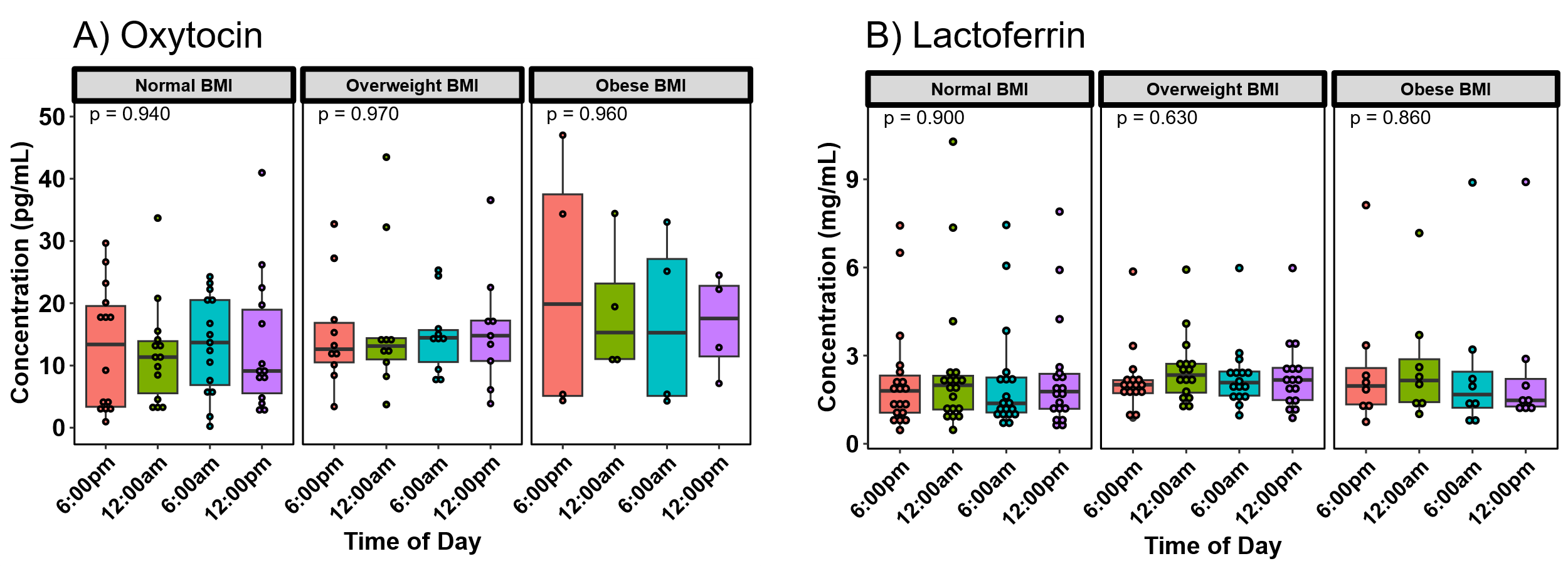
**Figure S7:** **Oxytocin and lactoferrin did not exhibit differences in concentration over a 24-hour period when divided by maternal BMI.** Concentrations of (A) oxytocin and (B) lactoferrin remained relatively stable over a 24-hour period and did not have significant differences between any of the time points (p > 0.05). Analysis for oxytocin included 15 sampling instances for normal BMI subjects, 10 sampling instances for overweight BMI subjects, and 5 instances for obese BMI subjects. Analysis for lactoferrin included 19 sampling instances for normal BMI subjects, 16 sampling instances for overweight BMI subjects, and 8 instances for obese BMI subjects. Group comparisons performed with Kruskal-Wallis, comparisons between two time points performed with Wilcoxon Rank Sum Test.


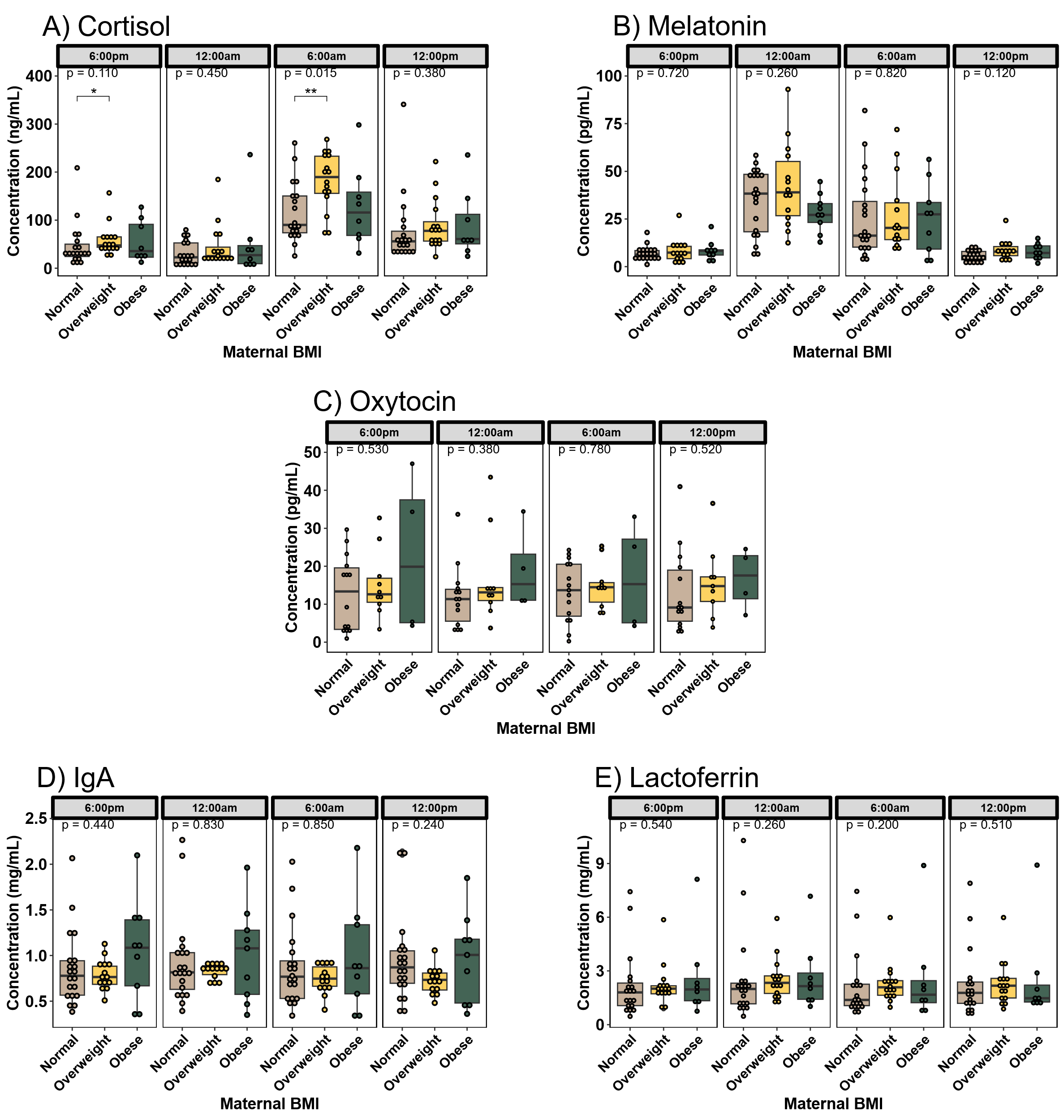


**Figure S8: Breast milk cortisol concentrations differ by maternal weight status when separated by time of day.** (A) Cortisol concentration in normal and overweight BMI subjects varied at the 6:00 pm and 6:00 am time points; no other differences noted in (B) melatonin, (C) oxytocin, (D) IgA, or (E) lactoferrin. Analysis for cortisol and lactoferrin included 19 sampling instances for normal BMI subjects, 16 sampling instances for overweight BMI subjects, and 8 instances for obese BMI subjects. Analysis for melatonin included 20 sampling instances for normal BMI subjects, 14 sampling instances for overweight BMI subjects, and 9 instances for obese BMI subjects. Analysis for oxytocin included 15 sampling instances for normal BMI subjects, 10 sampling instances for overweight BMI subjects, and 5 instances for obese BMI subjects. Group comparisons performed with Kruskal-Wallis, comparisons between two time points performed with Wilcoxon Rank Sum Test. * p < 0.05, ** p < 0.01, *** p < 0.001, **** p< 0.0001.


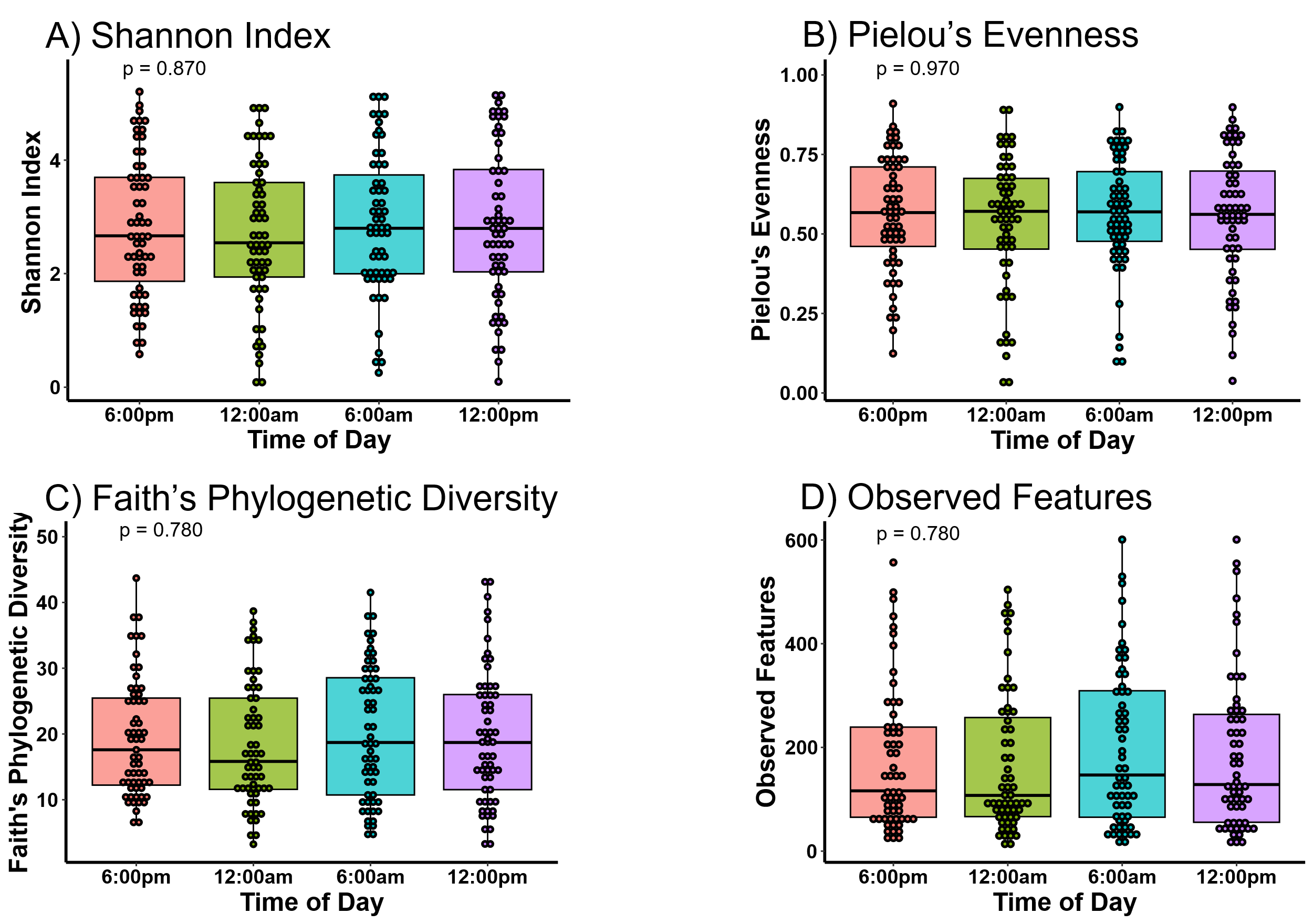
**Figure S9: Breast milk alpha diversity remained stable over 24-hour period.** No significant differences noted between time points for breast milk (A) Shannon Entropy, (B) Pielou’s evenness, (C) Faith’s Phylogenetic diversity, or (D) observed features (p > 0.05). Group comparisons performed with Kruskal-Wallis, comparisons between two time points performed with Wilcoxon Rank Sum Test.

**
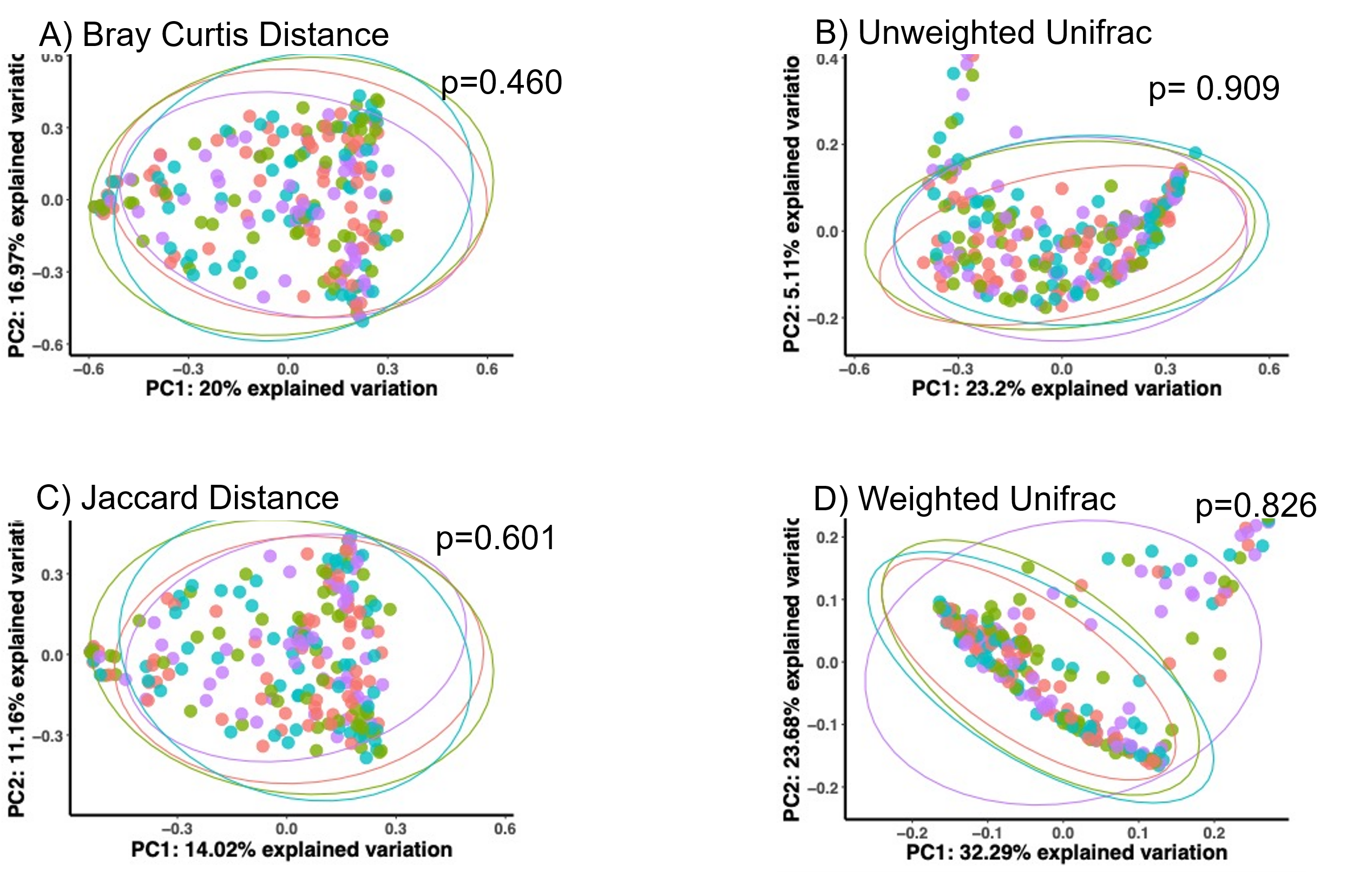
Figure S10: Breast milk beta diversity remained stable over a 24-hour period.** No significant differences noted for (A) Bray Curtis, (B) unweighted Unifrac, (C) Jaccard distance, or (D) weighted Unifrac metrics (p > 0.05). Comparisons for beta diversity performed using PERMANOVA.


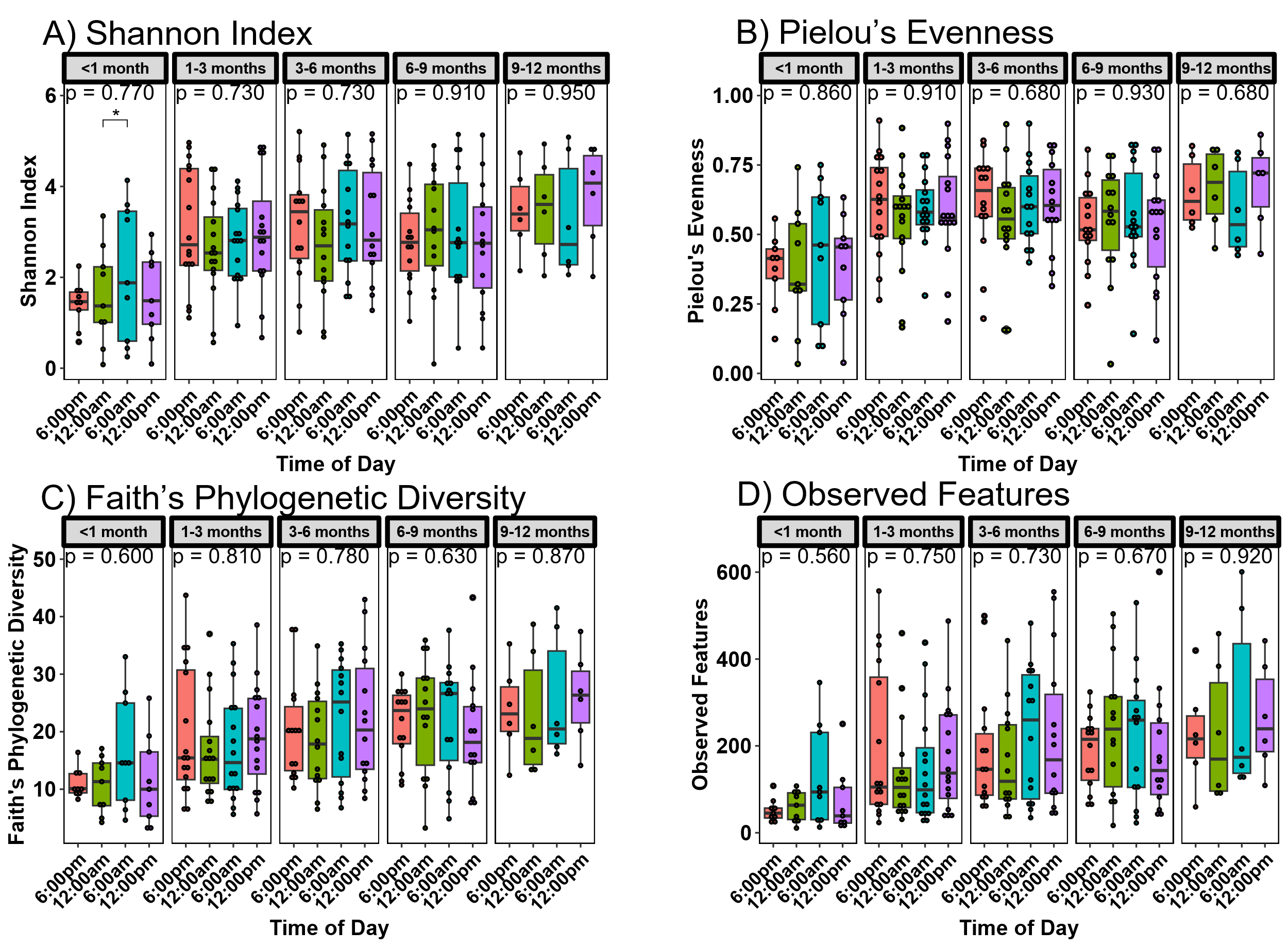


**Figure S11: Alpha diversity remains stable over 24-hours when separated by infant age.** (A) Shannon index was noted to be different between the midnight and 6:00 am time points in infants less than 1 month of age, but no other patterns emerged for (B) Pielou’s evenness, (C) Faith’s Phylogenetic diversity, or (D) observed features (p > 0.05). Microbiome analysis included 9 sampling instances <1 month, 16 sampling instances in 1-3 months, 14 instances in 3-6 months, 14 instances in 6-9 months, and 6 instances for 9-12 months of age. Group comparisons performed with Kruskal-Wallis, comparisons between two time points performed with Wilcoxon Rank Sum Test. * p < 0.05.


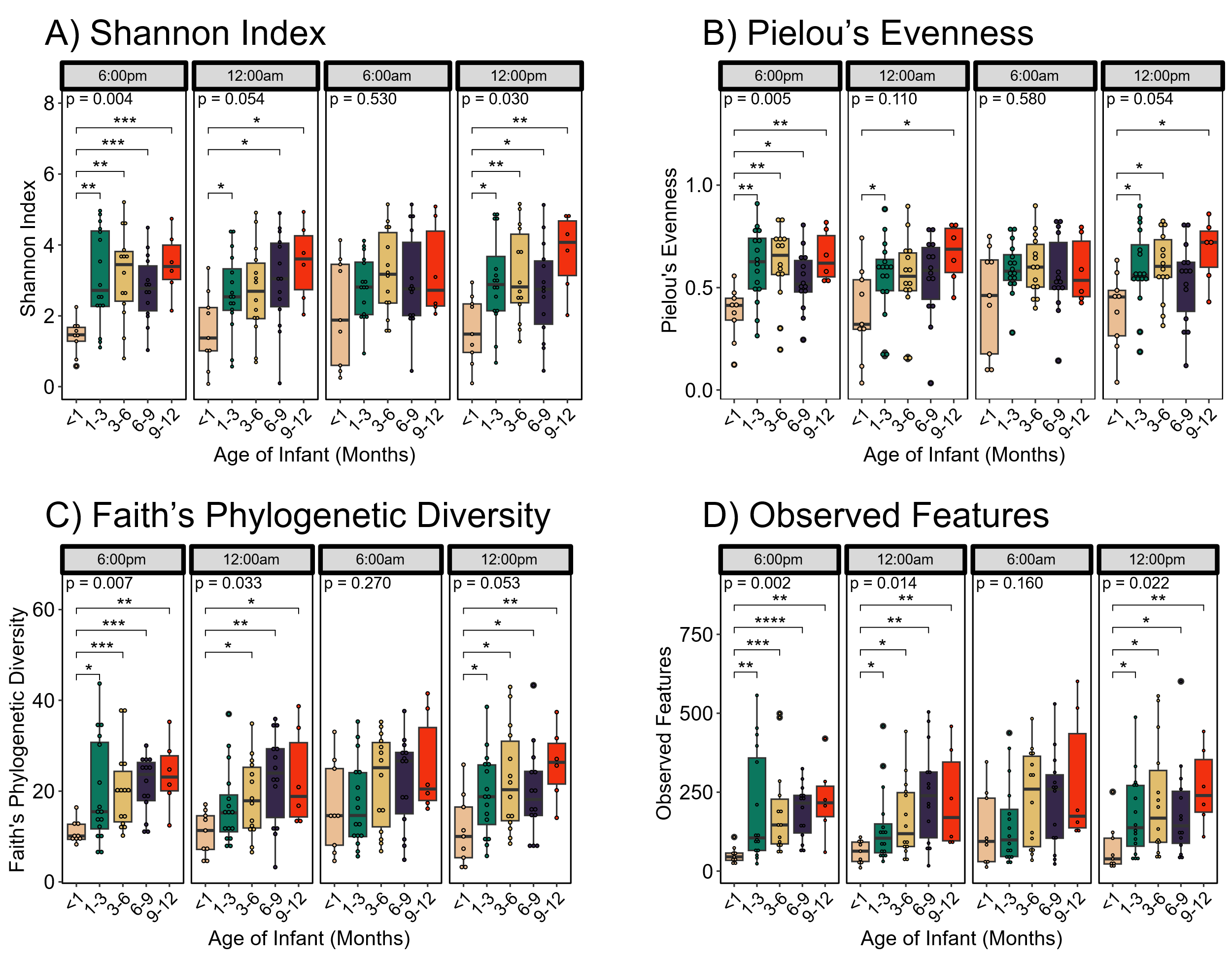


**Figure S12. Breast milk alpha diversity differs by infant age, except in the morning.** For subjects with infants <1 month of age, (a) Shannon Index, (b) Pielou’s evenness, (c) Faith’s Phylogenetic Diversity, and (d) Observed Features of the breast milk microbiome were significantly lower than the other age groups at each time point, but not at 6:00 am. Microbiome analysis included 9 sampling instances <1 month, 16 sampling instances in 1-3 months, 14 instances in 3-6 months, 14 instances in 6-9 months, and 6 instances for 9-12 months of age. Group comparisons performed with Kruskal-Wallis, comparisons between two time points performed with Wilcoxon Rank Sum Test. * p < 0.05, ** p < 0.01, *** p < 0.001, **** p< 0.0001.

**
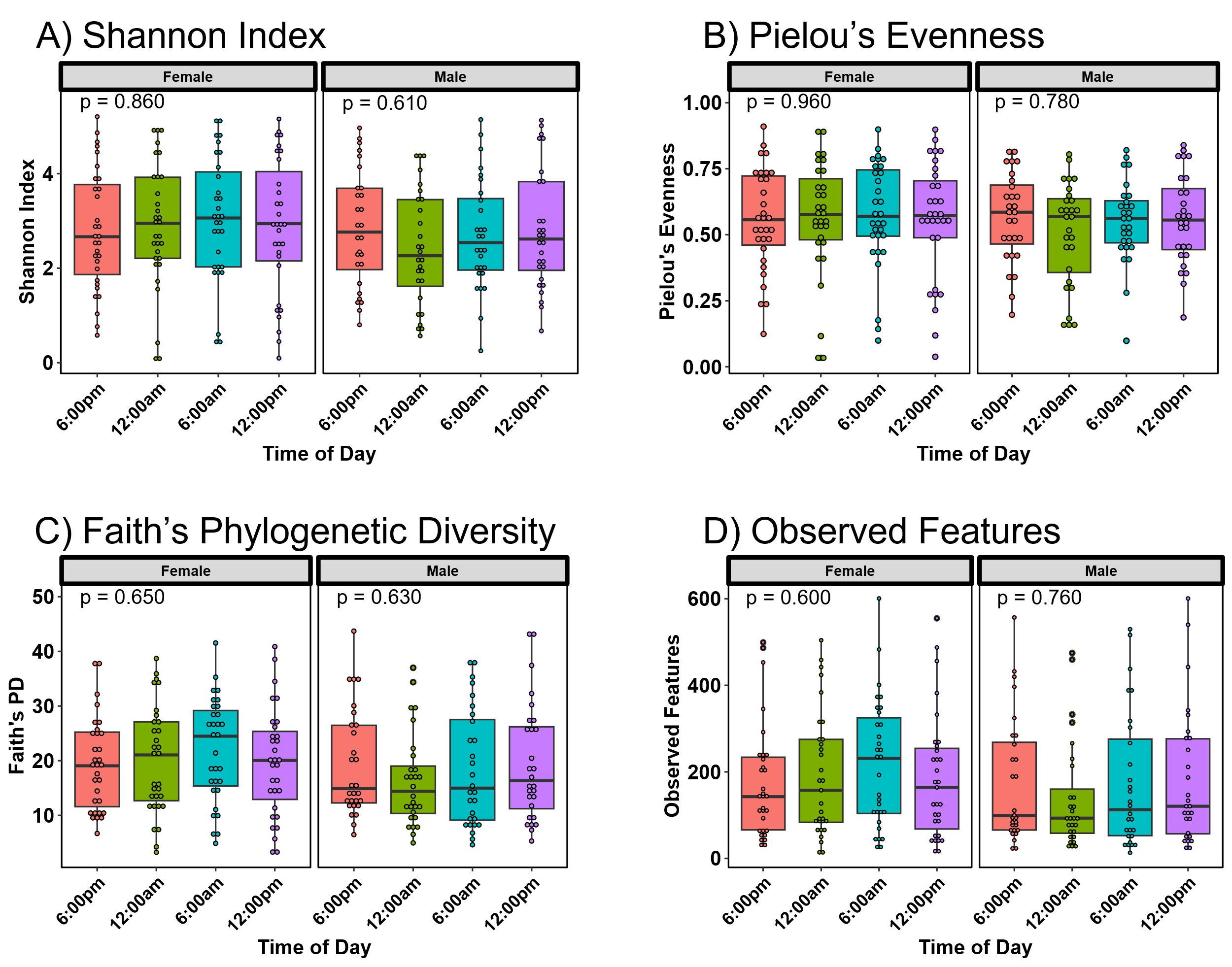
Figure S13: Alpha diversity of breast milk microbiome remained stable over a 24-hour period when looking separating subjects by having male or female infants.** No significant differences noted in (A) Shannon index, (B) Pielou’s evenness, (C) Faith’s Phylogenetic diversity, or (D) observed features over a 24-hour period when separating by maternal BMI (p > 0.05). Alpha diversity comparisons included 31 sampling instances for subjects with female infants and 28 sampling instances for subjects with male infants. Group comparisons performed with Kruskal-Wallis, comparisons between two time points performed with Wilcoxon Rank Sum Test.

**
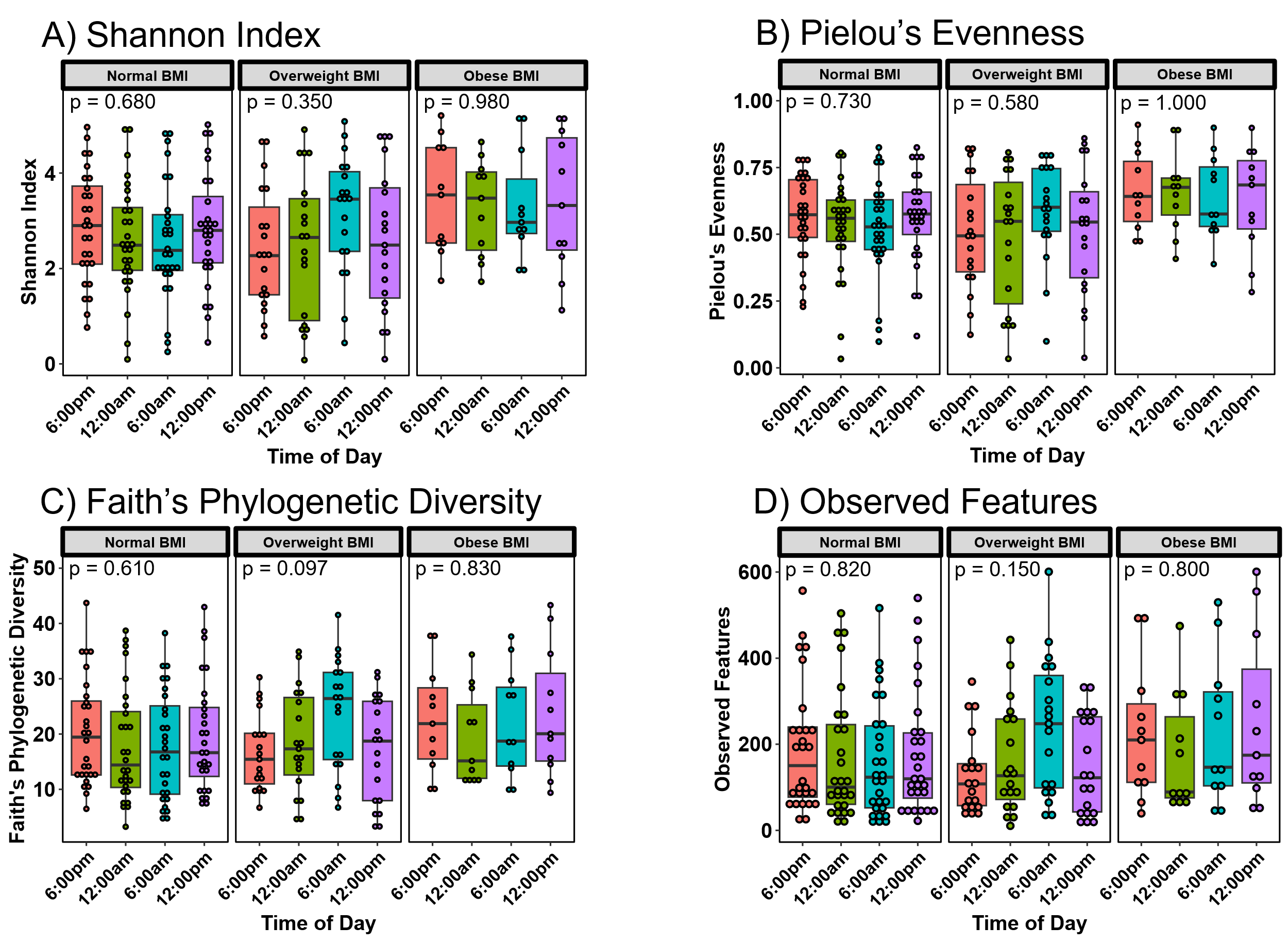
Figure S14: Alpha diversity in breast milk remains stable over 24-hour period when separating by maternal BMI.** No significant differences noted in (A) Shannon index, (B) Pielou’s evenness, (C) Faith’s Phylogenetic diversity, or (D) observed features over a 24-hour period when separating by maternal BMI (p > 0.05). Alpha diversity comparisons included 28 sampling instances for normal BMI subjects, 19 sampling instances for overweight BMI subjects, and 11 instances for obese BMI subjects. Group comparisons performed with Kruskal-Wallis, comparisons between two time points performed with Wilcoxon Rank Sum Test.

**Figure S15: An obese maternal BMI is associated with breast milk microbial ASV enrichment changes over a 24-hour period.** Only significantly different ASV abundances (p < 0.05) shown for subjects with an obese BMI; no significant differences were noted in ASV enrichment for subjects with normal or overweight BMIs. Microbiome analysis included 28 sampling instances for normal BMI subjects, 19 sampling instances for overweight BMI subjects, and 11 instances for obese BMI subjects. Comparisons of ASV enrichment performed using ANCOM with Bias Correction (ANCOM-BC).


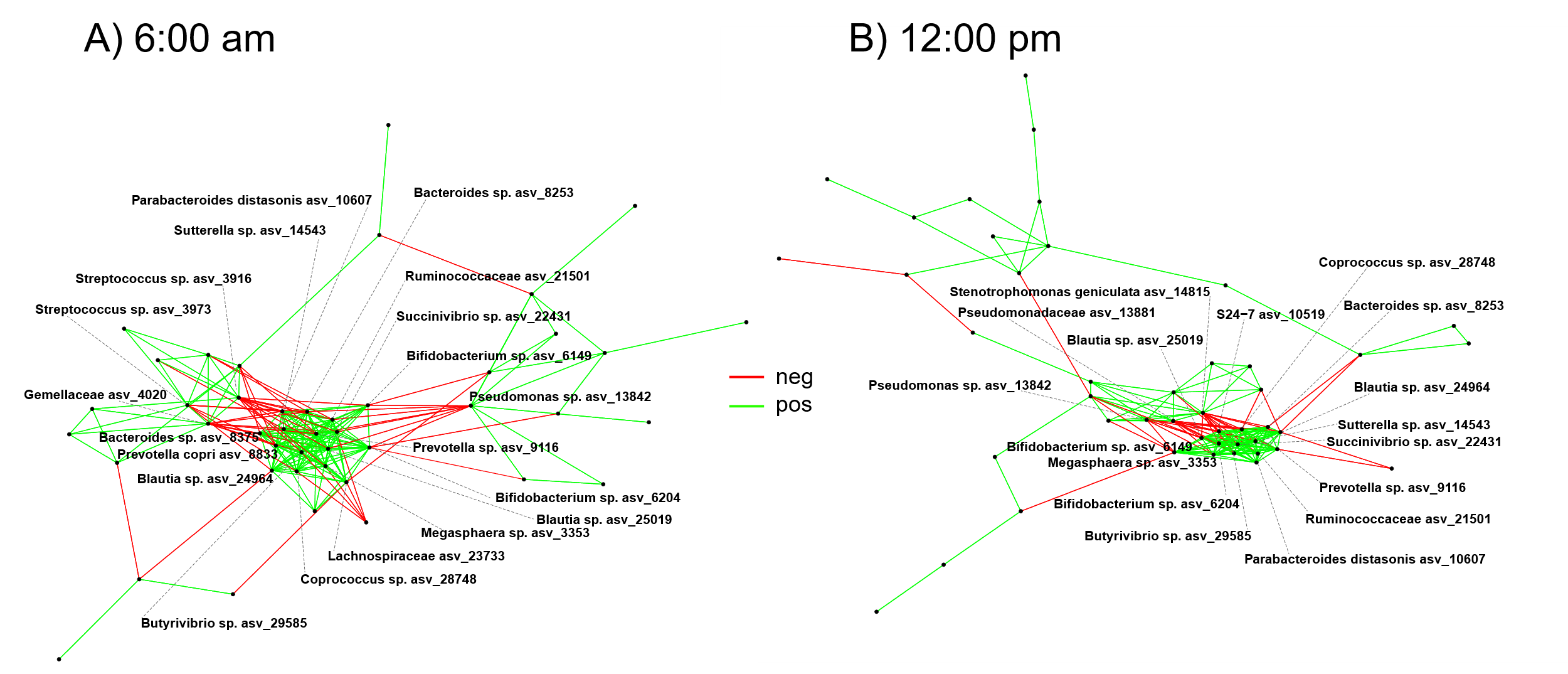

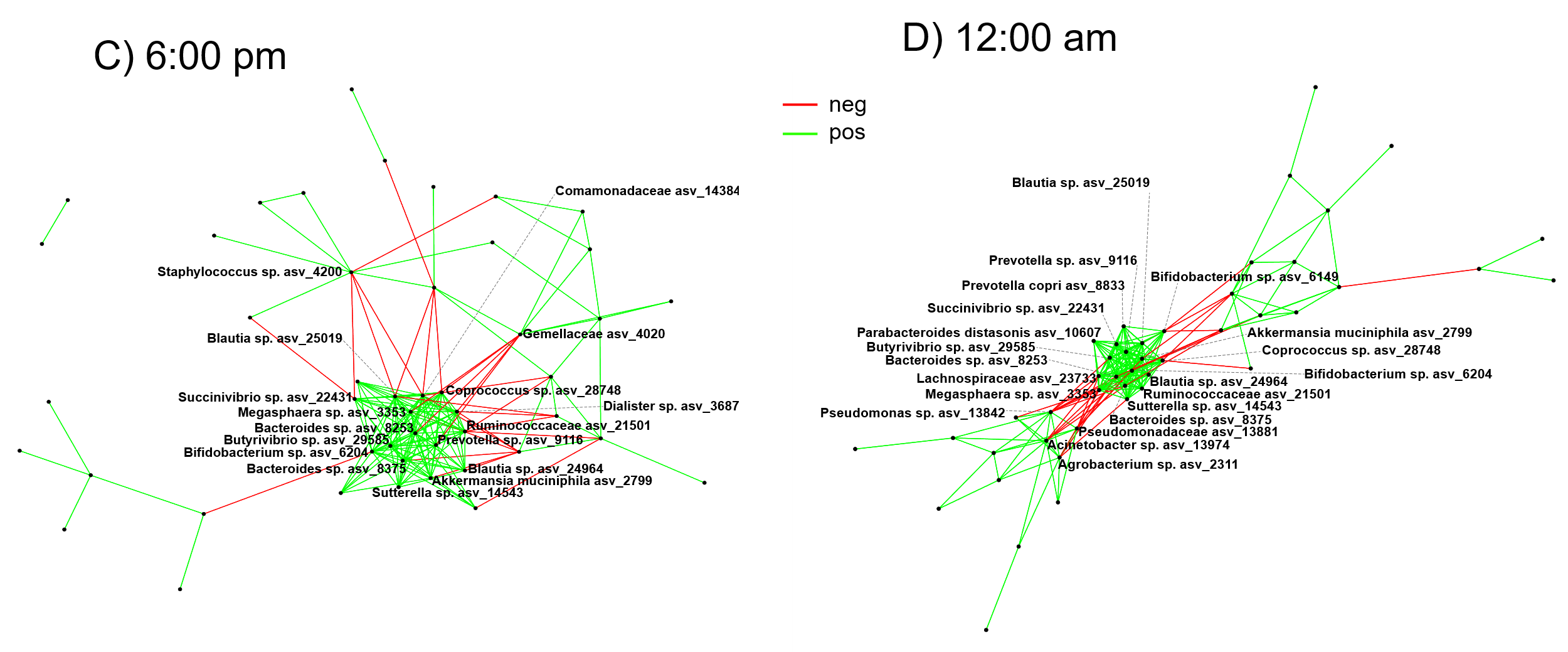


**Figure S16: Similar structure of breast milk microbial network over a 24-hour period.** Network analysis of the breast milk microbiomes at (A) 6:00 am, (B) 12:00 pm, (C) 6:00 pm and (D) 12:00 am did not have significantly different numbers of node connections when comparing time points (p > 0.05), but taxa with the most interconnectedness differed. Comparison of network metrics performed with Kruskal-Wallis across all time points, comparisons between two time points performed with Wilcoxon Rank Sum Test.
